# Discovery of bicyclic borane molecule B_14_H_26_

**DOI:** 10.1038/s42004-025-01409-1

**Published:** 2025-01-16

**Authors:** Xiaoni Zhang, Tomoko Fujino, Yasunobu Ando, Yuki Tsujikawa, Tianle Wang, Takeru Nakashima, Haruto Sakurai, Kazuki Yamaguchi, Masafumi Horio, Hatsumi Mori, Jun Yoshinobu, Takahiro Kondo, Iwao Matsuda

**Affiliations:** 1 The Institute for Solid State Physics (ISSP), The University of Tokyo, Kashiwa, Chiba 277-8581 Japan; 2 Institute of Innovative Research, Tokyo Institute of Technology, Yokohama, Kanagawa 226-8501 Japan; 3 Institute of Pure and Applied Sciences, University of Tsukuba, Tsukuba, Ibaraki 305-8573 Japan

**Keywords:** Materials chemistry, Chemical synthesis, Chemical bonding

## Abstract

The discovery of fullerene following the synthesis of graphene marked a paradigm shift in chemistry. Here, we report the discovery of biycycloborane, arising from the synthesis of borophane (hydrogen boride). Uniquely, this synthesis method involves a decomposition mechanism rather than traditional atom-by-atom assembly, marking an unique approach to constructing complex borane structures. The mass spectrometry unveiled that the stable molecule has a mass of 178 in atomic mass unit with a stoichiometry of B_14_H_26_. Optical spectra and simulations further evidenced its bicyclic structure, featuring fulvene-like heptagons or octagons. This borane molecule, analogous to cyclic hydrocarbons, adopts a unit configuration with a three-center two-electron (3c-2e) bonding, akin to diborane. The B_14_H_26_ molecule has been historically anticipated as a distant descendant of the dodecahedron borane, but it was born from the hydrogen boride sheet with a non-symmorphic symmetry. The discovery of biycycloborane expands the frontiers of boron chemistry, promising advancements in boron-based nanomaterials and beyond.

## Abstract (web summary)

Boron analogues of cyclic hydrocarbons have yet to be observed. Here, the authors isolate and characterize bicyclic borane molecule B14H26, advancing our understanding of borane chemistry.

## Introduction

The discovery of hydrocarbon molecules, such as benzene, has significantly impacted both fundamental chemistry research and industrial applications^1–3^. Boron, as an element analogous to carbon, can form various unique bonding configurations involving multicenter atoms and is expected to serve as a platform for developing advanced materials^4–8^. However, achieving stable structures of -“hydroboron”, so-called borane, has been challenging due to the electron deficiency character of boron. Traditional methods for constructing boranes often involve atom-by-atom assembly under strictly controlled conditions, making the discovery of new borane structures exceedingly difficult^9,10^. Recent theoretical and experimental advancements have increasingly focused on two-dimensional (2D) boron-based materials, such as borophene (B) and its hydrogenated counterpart, borophane (HB). These materials demonstrate remarkable stability due to the formation of three-center two-electron (3c-2e) bonds between boron and hydrogen atoms, a bonding configuration that is unique to boron’s electron-deficient nature^11–14^. While theoretical studies have proposed various potential structures for hydrogenated boron^15^, the experimental realization of such molecules remains an outstanding challenge. Consequently, the realization of zero-dimensional (0D) borane, akin to the well-known boron-hydride molecule diborane, has been eagerly anticipated as a significant breakthrough in boron chemistry^16–20^. Pioneering a method beyond the conventional atom-by-atom synthesis, the decomposition of higher-dimensional boron materials, such as borophane, emerges as a promising strategy to yield highly stable zero-dimensional (0D) boranes^12^. This innovative method offers a distinct pathway to access complex boron hydrides that have remained elusive through traditional synthetic techniques.

**Fig. 1:**
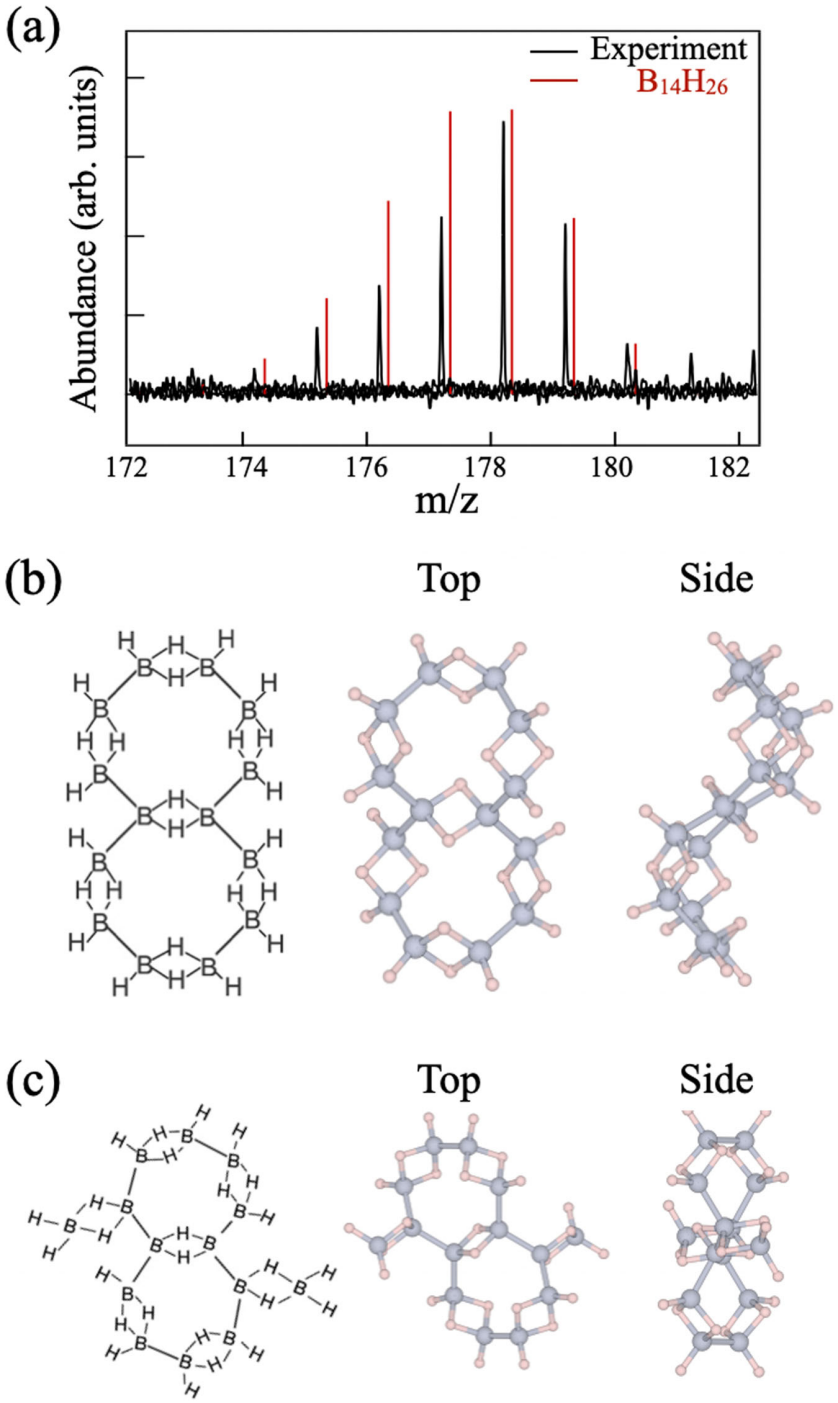
Detection of the B_14_H_26_ cluster and its molecular structures. **a** Mass spectrum at *m*/*z* = 178, where *m* and *z* represent the mass and charge number of ions, respectively. Simulated mass spectra of B_14_H_26_ are indicated by red bars. Atomic models of the B_14_H_26_ isomers: bicyclic structures of **b** octagons or **c** fulvene-like heptagons.

**Fig. 2:**
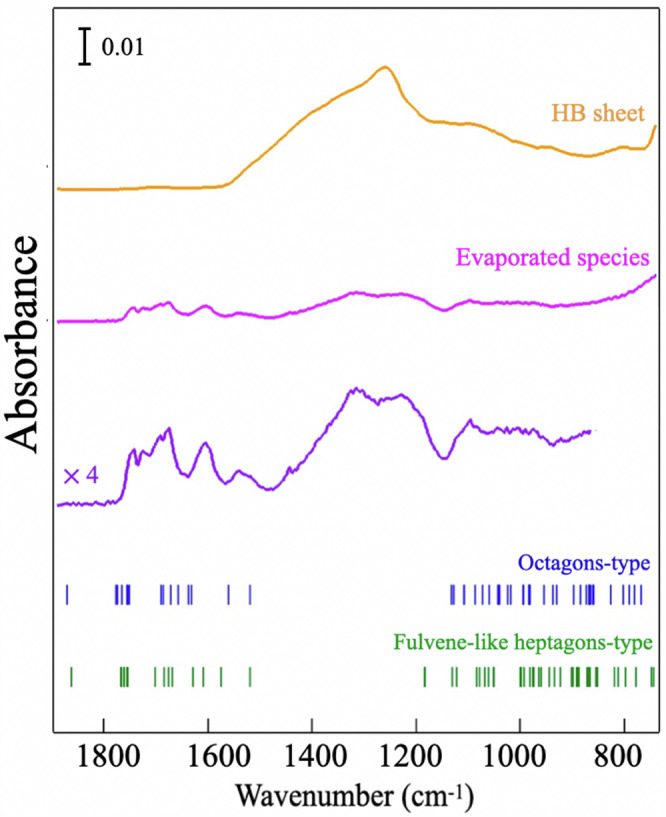
FT-IR spectra of HB sheets (yellow) and evaporate species (purple). Simulated patterns of vibrational modes for free B_14_H_26_ molecules with bicyclic structures of octagons (blue, Fig. 1b) and fulvene-like heptagons (green, Fig. 1c). The figure displays the simulated results of infrared-active vibrational modes.

Here, we report the discovery of an unexplored bicycloboraone molecule, B_14_H_26_, synthesized using chemical methods (Fig. 1a)^21^. Theoretical calculations revealed that the B_14_H_26_ molecule can adopt two distinct structures: a bicyclic structure of octagons (Fig. 1b) or a Fulvene-like heptagon structure, analogous to the carbon counterpart of fulvene-type heptalene (Fig. 1c). Based on our Fourier transform infrared absorption (FT-IR) results and theoretical calculations, we confirm that the synthesis method likely produces a mixture of these two isomers. The absorption spectra from Fig. 2 indicate features from both structures, suggesting that the sample contains a combination of octagon and heptagon forms. The B_14_H_26_ molecules are constructed of the B_2_H_2_ unit, which satisfies all valences via its 3c-2e bonding configuration, a feature well-known in diborane^11–14^. The molecular structure of B_14_H_26_ follows Wade’s rule^22–24^ and is a descendant of the mother dodecahedron borane. The generation number of B_14_H_26_ greatly exceeds those of existing borane clusters. This unexpected borane molecule was synthesized using the hydrogen boride (HB or borophane) atomic sheet.

## Results

### Identify the stoichiometry by mass spectrometry

The unusual molecule bicycloboraone, B_14_H_26_, was identified during the fabrication of free-standing sheets of hydrogen boride or “borophane”, which has attracted considerable attention in academic and technological fields^4,11–14^. Borophane sheets can be obtained by liquid exfoliation via the ion-exchange reaction of metal boride crystals that contain boron atomic layers^12,14^. When a Y*CrB*_4_ crystal is used as the mother material of the ion-exchange reaction, a product of the borophane sheets is composed of a two-dimensional framework of heptagons (seven membered-rings) and pentagons (five membered-rings) that are tiled non-symmorphically^11,13,25^. This reaction can also provide vaporized species that can be collected separately and examined sequentially. Approximately 15 mg of the solid compound can be isolated from 720 mg Y*CrB*_4_ (around 4 mMol) after synthesis. The vaporized compound was found to be stable at room temperature after dissolving in acetonitrile (Details in the Supplementary Information section 1: Fabrication of the bicyclic borane molecules). Along with pieces of the borophane sheet, mass spectrometry analysis detected notable peaks at *m*/*z* = 178 that were reproduced by mass spectrum simulations of B_14_H_26_ molecules, as shown in Fig. 1a.

Chemical structural formulas, as shown in Fig. 1b, c, were obtained by taking the B_2_H_2_ unit as a building block and modeling it from a two-dimensional boron framework of heptagons and pentagons in the Y*CrB*_4_ reactant (see the Supplementary information section 1: Fabrication of the bicyclic borane molecules, for details). The structure of the bicyclic borane molecule of octagons or fulvene-like heptagons is electrically neutral and satisfies all valences because of its three-center–two-electron (3c–2e) bonding configuration. It is of note that an octagon can be made from a bicyclic pentagon by breaking the contacting side. First-principles calculations^26^ were performed on the two types of molecules, which showed that their formation energies were almost identical. The optimized structures, which are presented in Fig. 1b, c, show that the individual boron atoms adopt the *sp*^3^-hybridized bonding configuration and the internal B-H-B bonds are arranged away from each other to minimize the steric hindrance in a molecule (The optimized structures are provided as Supplementary Data file 1 and 2). The chemical stoichiometry of B_14_H_26_ also allows a molecular structure with fulvene-like heptagon and octagon as the intermediate case, which is described in Supplementary information and provided as Supplementary Data file 4.

The molecular structure of the evaporated species was examined experimentally by FT-IR spectroscopy. The collected sample was installed in the measurement gas cell of the FT-IR spectrometer and then vaporized again by heating to 45 °C. The transmission FT-IR spectrum of the vapor is presented in Fig. 2. It is of note that water or solvent acetonitrile molecule does not contribute to the spectral features^27^. An FT-IR spectrum of the HB sheets synthesized from Y*CrB*_4_ crystals is also shown for comparison. The FT-IR spectrum of the HB sheets was measured by the attenuated total reflectance method.

### FT-IR characterization

The FT-IR spectrum of the HB sheets exhibits prominent features around 1350 c*m*^−1^ that are assigned to vibrational modes of the B-H-B bonds, as reported for the sheet^11,12,14,25,28,29^. In the FT-IR spectrum of the evaporated species, one can discern similar signals at corresponding wavenumbers. In addition, there are shoulder peaks in the regions above 1500 c*m*^−1^ and below 1200 cm^−1^ that are apparently different from the spectrum of HB sheets. To explore the origins of these unique vibrational features of the sample, the first-principles calculation was conducted on free B_14_H_26_ molecules of the octagons and the fulvene-like heptagons in vacuum (cf. Fig. 1b, c). The simulated frequencies of the vibrational modes are given in Fig. 2 as the reference. It is of note that two types of molecules belong to the *C*_*i*_ point group, and Fig. 2 displays only the modes that are infrared-active. The simulated patterns show vibrational modes in regions above 1500 cm^−1^ and below 1200 cm^−1^ that match the characteristic features in the FT-IR spectrum of the evaporated species. At the same time, the calculation does not provide any peak in the region between 1200 and 1500  cm^−1^, where the prominent peaks of the HB sheets are observed. It is, therefore, reasonable to consider that the FT-IR spectrum of the evaporated species contains contributions from both B_14_H_26_ molecules and pieces of HB sheets, which are consistent with those of mass spectrometry measurements of the evaporated samples (see the Supplementary information Section 3: Fourier transform infrared spectroscopy for details). The agreement between the experimental and simulated FT-IR patterns strongly supports the existence of the borane molecule B_14_H_26,_ which has the bicyclic structures of octagons and fulvene-like heptagons, as given in Fig. 1b, c.

### HOMO and LUMO calculation of the B_14_H_26_

**Fig. 3:**
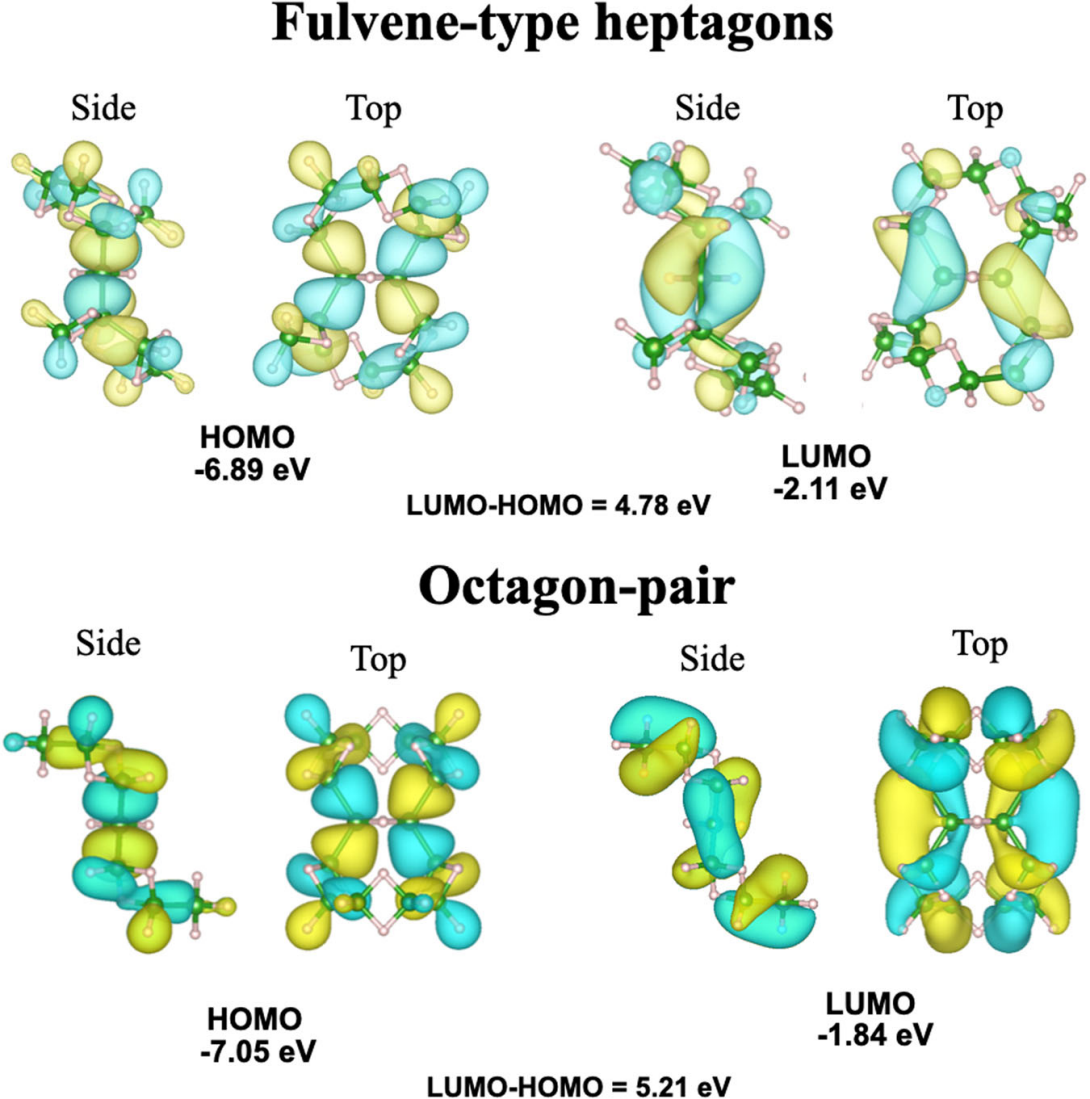
Calculated HOMO and LUMO energy levels for the predicted borane molecule B_14_H_26_ with fulvene-like heptagons or octagonal structure. The corresponding atomic structure is presented in Fig. 1. Colors correspond to the signs of the wave functions of orbitals.

The experimental observations reveal that the liquid exfoliation synthesis of HB sheets generates borane molecules, including the stable borane cluster B_14_H_26_. The molecular orbitals of the two structures of B_14_H_26_ are depicted in Fig. 3. In both cases, the highest occupied molecular orbital (HOMO) is of *σ*-bond type, whereas the lowest unoccupied molecular orbital (LUMO) has π^*^-orbital character. The energy level of the HOMO (LUMO) of the bicyclic octagon structure is located at -6.89 (-2.11) eV with respect to the vacuum level, whereas the HOMO (LUMO) of the bicyclic fulvene-like heptagon structure appears at -7.05 (-1.84) eV.

Boranes, B_*x*_H_*y*_, exhibit various structures that are distinct from those of hydrocarbons because of the multicenter bonding configurations of boron atoms^15–20,30^. B_*x*_H_*y*_ compounds range from the simple molecule borane (BH_3_) and well-known diborane (B_2_H_6_) to the abundant clusters of boron hydride and the various atomic sheets of boraphene^4–8,31,32^. As a counterpart of cyclic hydrocarbons, cyclic borane molecules have been investigated theoretically by replacing unsaturated (-CH=CH-) bonds with 3c-2e bonds (-BH_2_B-)^33^. Based on an analogy of the well-known Hückel model, the “Hückeloid” model was developed to discuss the energy of cyclic borane molecules^34,35^. The Hückeloid model provides deeper insights explaining the electronic properties and stability of B_14_H_26_. By highlighting how the 3c-2e bonding scheme leads to a delocalized electron system similar to aromaticity in hydrocarbons, this model offers a deeper understanding of the molecule’s stability and small HOMO-LUMO gap, both of which are critical for its reactivity and potential applications in boron-based nanomaterials. By integrating this model into the discussion, we reinforce the theoretical foundation of our findings and align it with our observations. Further details on the application of the Hückeloid model to both the octagonal and fulvene-like heptagonal isomers are provided in the Supplementary Information Section 7: The Hückeloid model for cyclic borane molecules.

## Discussion

Traditionally, B_*x*_H_*y*_ boranes have been synthesized using direct methods such as hydroboration or reductive coupling of boron halides, processes that typically require high temperatures, pressures, or specialized reagents. These methods often yield simpler borane structures, such as B_10_H_14_ (decaborane) or ${[{{{{\rm{B}}}}}_{12}{{{{\rm{H}}}}}_{12}]}^{2-}$ (dodecaborate)^10,36–38^. In contrast, the borane material B_14_H_26_ was serendipitously generated during the evaporation of atomic sheets of hydrogen boride as they formed. This discovery mirrors the identification of fullerenes, which were first recognized following the evaporation of graphite sheets by laser ablation. Remarkably, the discovery of the previously unknown “large" borane molecule B_14_H_26_ was not achieved through conventional synthesis but rather via a decomposition mechanism during the preparation of borophane sheets. The decomposition process, facilitated by an ion-exchange reaction under mild conditions (e.g., room temperature and low pressure), involves the exfoliation of boron layers with a framework of heptagons and pentagons, followed by proton doping. The protons selectively cleave B-B bonds in the borophane sheets, particularly within the strained 5,7-rings. This bond cleavage is crucial for generating reactive boron-hydride intermediates. As these intermediates form, they undergo rearrangement or condensation, leading to the formation of B-H-B bonds that stabilize the bicyclic B_14_H_26_ structure. This discovery introduces a distinct paradigm, wherein complex borane structures can emerge from the breakdown of larger boron-containing materials, contrasting sharply with traditional synthetic methods that typically involve atom-by-atom construction of borane clusters in a controlled environment. Cyclic materials are expected to show unique topological properties that could reveal unexplored branches in organic chemistry. Moreover, the restriction of the cyclic structure of B_14_H_26_ may provide possible intermediate states beneficial for catalytic reactions.

Varieties of borane and derivatives have been found historically, and the present molecular structures follow the stream of boron chemistry. Classifications of borane clusters have been made theoretically and Wade’s rule^22–24^ has been widely used to explain structural relationships in terms of the number of atoms and valence electrons. In brief, a structure begins with a polyhedron, such as a tetrahedron or an octahedron, with a boron atom at the vertex and each boron atom is bonded with one hydrogen atom. Wade’s rule states that the stable structure depends on the sum of the numbers of boron atoms (i), hydrogen atoms (j), and electrons that were not involved in the BH bonds at the vertex (k). Borane clusters are expected to be generated when i + j + k = 2i + 2 (*closo*-structure), 2i + 4 (*nido*-), 2i + 6 (*arachno*-), or 2i + 8 (*hypo*-). The individual structures can be represented by chemical formulas of B_*m*_H_*m*+2_ for *closo*-, B_*m*_H_*m*+4_ for *nido*-, B_*m*_H_*m*+6_ for *arachno*-, and B_*m*_H_*m*+8_ for *hypo*-. As the ratio of the boron atoms decreases along this series, the borane cluster generally becomes more unstable. For pristine borane, to date, there has been no report of the *hypo*-structure.

**Fig. 4:**
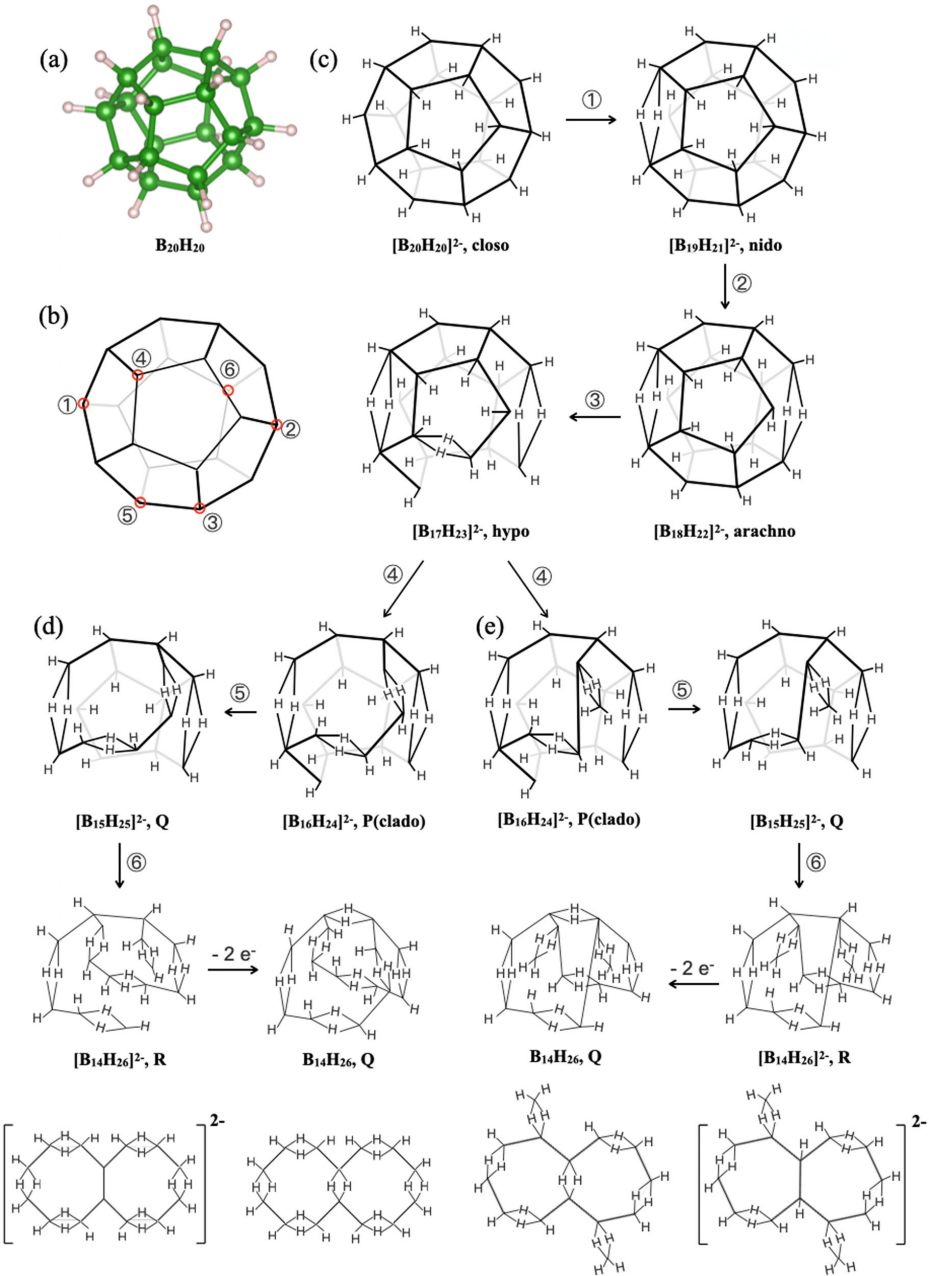
The derivation pathways for 2 types of B_14_H_26_. **a** The dodecahedron structure of the B_20_H_20_ cluster. **b** Schematic diagram of the B_20_ cluster; the six distinct boron atoms are labeled by number. By removing six vertex boron atoms, the **c** *Q*-structure (B_*m*_H_*m*+12_-type) B_14_H_26_, **d** octagonal-pair isomer, and **e** fulvene-like bicyclic borane molecule can be obtained.

A search of the numerical sequence of the borane series has been conducted theoretically and the B_14_H_26_ molecule was listed as a B_*m*_H_*m*+12_-type structure^24^. Borane was labeled as the *Q*-structure, and its real atomic configuration has been questioned. The molecular structure of B_14_H_26_ follows Wade’s rule^22–24^, and it is one of the sequential borane molecules derived by removing boron vertices from the dodecahedron structure, as shown in Fig. 4. The structure of B_14_H_26_ presented in Fig. 1 can be geometrically obtained by removing the appropriate six sites of vertex boron atoms and the number of valence electrons can be satisfied by generating seven -BH_2_B- bonds in the 3c-2e scheme. The conformity of the B_14_H_26_ structure to Wade’s rule validates the concerted endeavor within our field to study boron materials. The present results mark a significant step forward in our understanding of boron materials. The finding of the *Q*-structure (B_*m*_H_*m*+12_-type) before a report of the *hypo*- (B_*m*_H_*m*+8_) or *klado*- (*B*_*m*_*H*_*m*+10_) borane structure is likely due to the lack of a universal synthetic method for exploring borane molecules.

From countless candidate predicted structures, our research has conclusively demonstrated the discovery of an unexplored borane molecule, B_14_H_26_, with a bicyclic structure comprising either octagons (Fig. 1b) or fulvene-like heptagons (Fig. 1c). These structures are confirmed to be stable through first-principles calculations. The innovative approach of fabricating this cyclic borane via the fragmentation of boron sheets introduces a distinct methodology that is both simple and energy-efficient, utilizing liquid exfoliation at room temperature. This study not only expands the scope of boron chemistry by presenting a previously unexplored type of stable borane but also opens the door for future research into energy-efficient synthetic techniques. Furthermore, the ease of this method suggests its potential applicability in the scalable production of complex boron-based materials, offering a promising platform for developing advanced functional materials in nanotechnology and other related fields.

The structural stability of B_14_H_26_, as explained by Wade’s rule, can be compared with other well-known boranes, such as B_10_H_14_ (decaborane) and ${[{{{{\rm{B}}}}}_{12}{{{{\rm{H}}}}}_{12}]}^{2-}$ (dodecaborate)^10,36–38^. These molecules exhibit traditional, symmetrical cage-like structures stabilized by 3c-2e bonding configurations, which are central to their stability. B_14_H_26_, on the other hand, features a unique bicyclic structure with larger octagon and fulvene-like heptagon motifs, providing a unique framework that deviates from the more compact geometries observed in decaborane and dodecaborate.

Recent studies in boron chemistry emphasize the importance of delocalized electron systems in stabilizing larger boron clusters. For instance, delocalization phenomena observed in complex boron structures have been shown to enhance stability^39^. Unlike traditional boranes that often require external stabilization^40^, B_14_H_26_ achieves stability intrinsically through its unique bonding configuration, making it a promising candidate for further exploration of larger boron frameworks.

In addition, recent advancements in boron nanomaterials^41,42^ emphasize tuning electronic properties for specific applications. B_14_H_26_ small HOMO-LUMO gap and delocalized *π*-electron system suggest potential uses in areas like energy storage and catalysis. By comparing B_14_H_26_ with established boranes and recent developments in boron chemistry, we highlight its structural uniqueness and its potential for future applications in advanced materials, such as hydrogen storage by the high hydrogen content and catalysis based on its electron-deficient framework.

## Methods

### Synthesis on molecule B_14_H_26_

Yttrium chromium boride powder (Y*CrB*_4_, 700 mg, synthesized by arc-melting in the laboratory) and a cation-exchange resin (60 ml, 0.5-1 mm, Amberlite IR120B H HG, Organo Corp., Tokyo, Japan) were mixed in acetonitrile solvent (200 ml, 99.5%, JIS special grade, FUJIFILM Wako Pure Chemical Corp, Osaka, Japan) at room temperature, followed by stirring at the atmospheric pressure in an Argon atmosphere. After 3 days of stirring, the remaining Y*CrB*_4_ and resin were removed from the solution via a membrane filter (0.1 μm, Omnipore Membrane Filters, Merck Millipore, Billerica, MA). The filtrate, which was a yellow dispersion, was heated under a vacuum at 0.1 MPa and 80 ^∘^C using a water bath, and the vaporized filtrate was collected. The step-by-step synthesis can be found in Supplementary information section 1: Fabrication of the bicyclic borane molecules.

### Characterization

Mass spectra were recorded by a mass spectrometry system (JEOL JMS-AX500) with a field desorption probe in the positive mode. The measurements were conducted on the solution samples containing the vaporized species in acetonitrile solvent. Experiments of FT-IR spectroscopy was made by two types of the measurement set-up, depending on samples. FT-IR spectra of gas samples were recorded with JASCO FT/IR-6000 Series in transmission geometry, while those of sheets were with BRUKER ALPHA II FT-IR spectrometer in reflection geometry.

### Details of the first-principles calculation

The Python package “Psi4 1.9.1" was used to calculate the optimized molecular structure, molecular orbitals, and vibrational properties^43^. The Becke Three Parameter Exchange and Lee-Yang-Parr Correlation Functional (B3LYP) were chosen as the exchange-correlation functional^44^. The Gaussian basis set 6-311g(d,p) was used to describe the electronic structures. The molecular geometry was optimized under Gaussian-level criteria. To obtain thermally stability or thermal relaxation structure, the molecular dynamics (MD) simulations were performed via OpenMX v.3.9^45^, which is the first-principles code based on density functional theory (DFT), norm-conserving potential, and pseudo-atomic localized basis functions^46–48^. All the MD calculations via OpenMX were performed utilizing a generalized gradient approximation (GGA) and the basis sets of H6.0-s2p1 and B7.0-s2p2d1^49^. The regular mesh of 260 Ry in real space was used for the numerical integration and for the solution of the Poisson equation. The temperature was kept at 300 K based on the velocity scaling method for 250 fs. The time interval was set to be 0.5 fs^50^ (an electronic temperature of 300 K is to count the number of electrons by the Fermi-Dirac function). The initial molecular structures were placed in the unit cell with cubic lattice parameters, ensuring that the minimum distance intersecting the periodic boundary between the atoms is greater than 14 Å.

## Supplementary information

Supplementary Information

Description of Additional Supplementary Data Files

Supplementary Data 1

Supplementary Data 2

Supplementary Data 3

Supplementary Data 4

## Data Availability

The data that support the findings of this study are available from the corresponding author upon reasonable request. The optimized structures for molecules B_14_H_26_ in this study are provided as the Supplementary Data 1–2. The intermediate case, B_14_H_26,_ with fulvene-like heptagon and octagon molecule structure, is described in the Supplementary Information, and the corresponding optimized structure is provided in the Supplementary Data 4. An optimized structure of B_8_H_14_ molecule with a pair of pentagons, also described in Supplementary Information, is provided in Supplementary Data 3.
