# Supplementary material for: Discovery of bicyclic borane molecule B14H26
Source: Commun Chem. 2025 Jan 16;8:14. doi: 10.1038/s42004-025-01409-1 (PMC11739403; doi:10.1038/s42004-025-01409-1)
Supplement: Supplementary file 1 — Supplementary Information [file 42004_2025_1409_MOESM1_ESM.pdf]

Supplementary Information: Discovery of bicyclic borane molecule $B_{14}H_{26}$

Xiaoni Zhang,† Tomoko Fujino,† Yasunobu Ando,‡ Yuki Tsujikawa,† Tianle Wang,† Takeru Nakashima,‡ Haruto Sakurai,† Kazuki Yamaguchi,† Masafumi Horio,† Hatsumi Mori,† Jun Yoshinobu,† Takahiro Kondo,¶ and Iwao Matsuda*,†

†*The Institute for Solid State Physics (ISSP), The University of Tokyo, Kashiwa, Chiba 277-8581, Japan*

‡*Institute of Innovative Research, Tokyo Institute of Technology, Yokohama, Kanagawa 226-8501, JAPAN*

¶*Institute of Pure and Applied Sciences, University of Tsukuba, Tsukuba, Ibaraki 305-8573, Japan*

E-mail: imatsuda@issp.u-tokyo.ac.jp

Contents

1 Fabrication of the bicyclic borane molecules **2**

2 Details on mass spectrometry **3**

3 Fourier transform infrared spectroscopy **10**

4 Stability of borane molecules in acetonitrile solution **11**

5 Structure information from X-ray diffraction and NMR spectroscopy **12**

6 **Bicyclic borane molecule with a octagon and a fulvene-like heptagon** **17**

7 **The Hückeloid model for cyclic borane molecules** **17**

8 **Cohesive energies analysis on the bicyclic $B_{14}H_{26}$ molecule** **23**

9 **Methods** **23**

1 Fabrication of the bicyclic borane molecules

The molecule $B_{14}H_{26}$ was discovered after collecting vaporized species during synthesis of the hydrogen boride (HB) sheets (Fig.S1(a)). The sheet synthesis has been made through liquid exfoliations of $YCrB_4$ crystals (Fig.S1(b)), based on the ion exchange reaction:[1–5]

$$n\mathrm{YCrB_4} + 4n\mathrm{H^+} \rightarrow n\mathrm{Y^{(4-x)+}} + nCr^{\mathrm{x}+} + 4n\mathrm{HB}.$$

The mother material of a $YCrB_4$ crystal was prepared by the arc melting method with a composition ratio of Y: Cr: B = 1: 1: 4.[2,6,7] Subsequently, the as-fabricated crystals were divided into pieces smaller than 1 mm. The quality of $YCrB_4$ was assessed using X-ray diffraction (XRD), revealing high crystallinity compared to the the simulated pattern of the crystal structure (Fig.S2).

To make a collection of the vaporized boron species, we made a protocol as illustrated in Fig.S3. At first, 700 mg of the $YCrB_4$ crystals were mixed with 60 ml cation ion exchange resin beads (0.5-1 mm, Amberlite IR120B H HG, Organo Corp., Tokyo, Japan) in 200 ml acetonitrile solvent (99.5%, JIS special grade, FUJIFILM Wako Pure Chemical Corp, Osaka, Japan). The ion-exchange reaction proceeded by stirring the solution in the Ar atmosphere at room temperature, Fig.S2(a), and was promoted by adding 2 ml of 1 M hydrochloric acid (HCl). It is of note that 1 ml of 1 M hydrochloric acid (HCl) was added for every 2 mmol of $YCrB_4$. After 7 days of the reaction, the solution was filtered by a 0.1 μm pore filter (Omnipore Membrane Filters, Merck Millipore, Billerica, MA). The filtrate, appeared in yellow, was subsequently heated under vacuum at 0.1 MPa and 80°C using a

water bath, as shown in Fig.S3(b). The heating process induces vaporization of the filtrate and the vaporized species were collected in the connected vessel. At room temperature, the vessel contained a yellow-colored solution, shown in Fig.S3(c), that contains borane molecule and acetonitrile solvent. We confirmed that the pure acetonitrile solution resulted in a transparent liquid following the same heating procedure. Upon heating the yellow-colored solution shown in Fig. S3(c) to 40°C, the dried specie remained as a solid at the bottom of a vessel, Fig.S3(d). We were able to isolate approximately 10 mg of the solid compound of species, synthesized from 720 mg $YCrB_4$ (around 4 mMol).

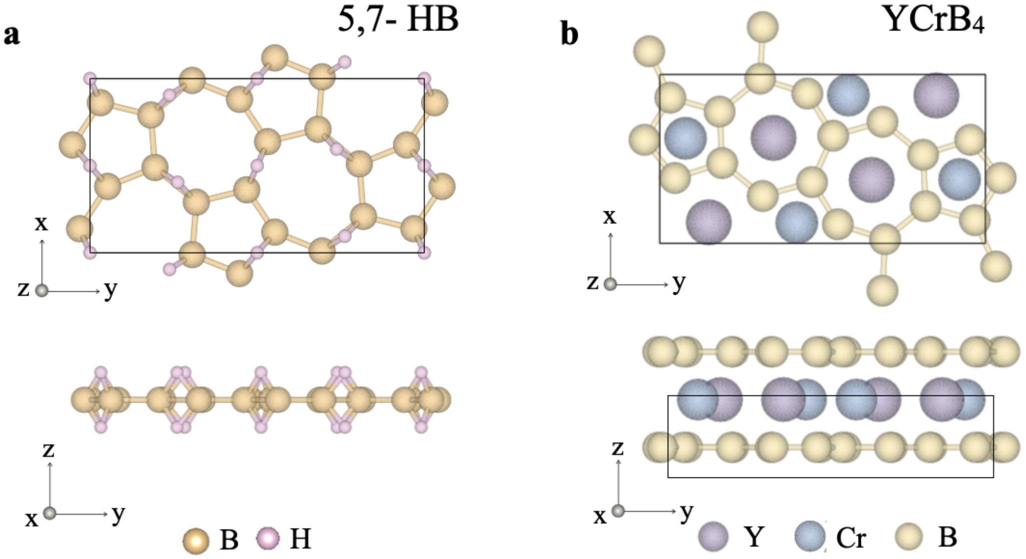

Figure. S 1: Atomic structures of (a) the hydrogen boride (HB) sheet and (b) the mother material, $YCrB_4$ crystal.[8]

2 Details on mass spectrometry

The measurements were conducted on the solution samples containing the vaporized species in acetonitrile solvent, as referred to Figure. S3(c). During the experiment, two types of the mass spectra were recorded, as shown in Fig.S4. It is of note that assignments of the borane peaks were made by the mass spectrum simulator (Prot pi[9]). The non-borane

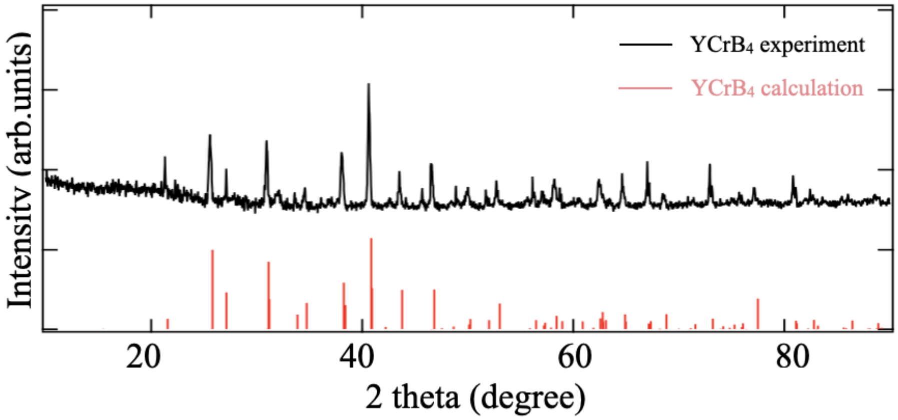

Figure. S 2: A comparison between an experimental X-ray diffraction (XRD) pattern for the $YCrB_4$ crystal (black) and the simulated one (red)[8]

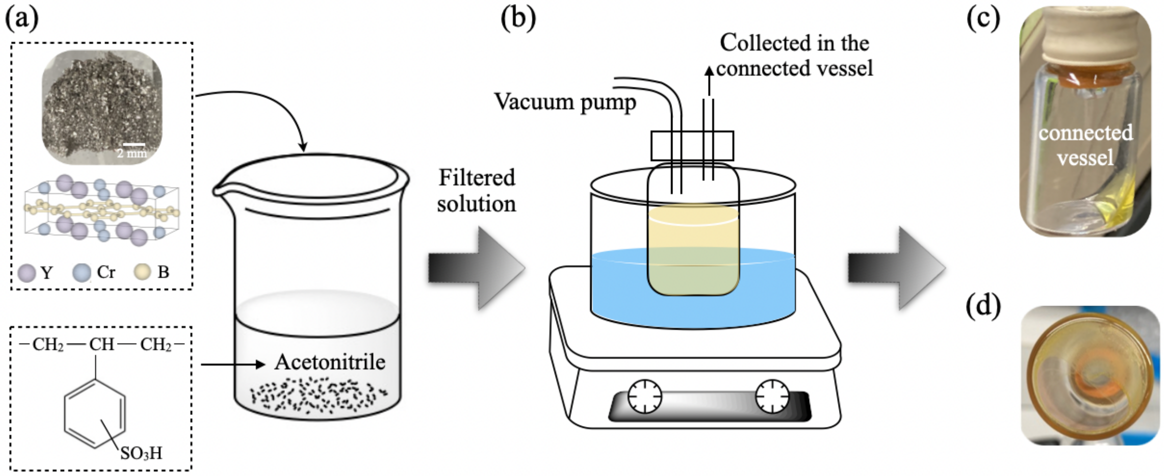

Figure. S 3: A collection procedure of vaporized boron species: (a) The ion-exchange reaction process and (b) the collection process by heating the filtered solution. (c) The resulting solution sample, collected in a vessel, and (d) the dried sample, remained at the bottom of a vessel, after heating the solution (c).

peaks correspond to solvent molecules, acetonitrile (m/z $\sim$ 41), and impurities, such as boric acid at (m/z $\sim$ 62). One type of the spectra contains a single peak for the $B_{14}H_{26}$ molecule at m/z $\sim$ 178 as shown in Fig.S4(a). In addition to the $B_{14}H_{26}$, a prominent peak at m/z 192 was observed in the mass spectrometry data. This peak likely corresponds to a derivative of $B_{14}H_{26}$ with the addition of a $-BH_3$ fragment. The mass increase of approximately 14 units is consistent with the molecular weight of the $-BH_3$ group, a known fragment in borane chemistry. Such an addition could occur during the vaporization or ionization processes, where reactive $B_{14}H_{26}$ species interact with $-BH_3$ to form a stable complex. This behavior has been documented in other borane clusters, where boron-hydride fragments readily attach to form larger or more complex structures.[10] The $B_{14}H_{26}$ molecule corresponds to a bicyclic structure of octagons (Fig.1(b)) or fulvene-like heptagons (Fig.1(c)) , as described in the main paper. The other type of the spectra, given in Fig.S4(b), exhibits multiple borane peaks up to m/z $\sim$ 1650 and it is likely that most of the mass patterns are ascribed to cracking components of a large borane molecules. The prominent peak, labeled A, was found at m/z $\sim$ 101. It is ascribed to the B_8H_{14} molecule, confirmed by matching with the simulation (Fig.S5(a) and Supplementary Data file 3). A periodic pattern, labeled B, was found at m/z = 1000 - 1650 with a periodicity of Δm/z = 74, consisting of paired peaks separated by Δm/z = 14. The mass period can be corresponded to the borane unit of B_6H_8 and the mass separation to the unit of BH_3. The results of mass spectrometry leaves a potential to discover unexpected borane molecules by the fabrication procedure, described in Fig.S3.

We now recall the B_8H_{14} molecule, shown in Fig.S4(b) and Fig.S5(a), to deepen the discussion. Based on the Wade's rule, the chemical formula indicates that the molecule belong to *arachno*-B_8H_{14} that can be derived from *closo*-$B_{10}H_{12}$ or *closo*-$[B_{10}H_{10}]^{2-}$. One can consider the molecular structure based on the crystal structure of the HB sheet that has a boron network of pentagons and heptagons. Among possible isomers,[11–15] the most appropriate structure can be a bicyclic molecule of pentagons, as depicted in Fig.S5(b).

Following Wade's rule, one can also derive *arachno*-B_8H_{14} from *closo*-$[B_{10}H_{10}]^{2-}$ by two steps, as illustrated in Fig.S5(c). A structural optimization of the B_8H_{14} molecule by calculation results in the flat bicyclic molecule, as shown in Fig.S6 (a). The molecular orbitals of the B_8H_{14} are also calculated and depicted in the Fig.S6 (b,c). The energy levels of HOMO (LUMO) for the B_8H_{14} are located at -6.86 (-2.80) eV with referred to the vacuum level. It is of note that the molecular structure is not in a list of the possible bicyclic molecules, examined recently.[16] The B_8H_{14} molecule with a pair of pentagons is likely a $B_{14}H_{26}$ molecule that was discovered by the present research.

It is intriguing to observe prominent peaks at m/z $\sim$ 178 and 101 in the mass spectra of sample-1 and sample-2 types, respectively. As described in the main paper, the peak at m/z $\sim$ 178 corresponds to a possible isomer with a heptagon-based bicyclic structure. It is, thus, inferred that appearance of the mass pattern types, Fig.S4 (a) and (b), depends on dominant formation of the bicyclic heptagon- and pentagon-types, respectively. Optimized regulations of the method could potentially enable the selective preparations of the borane molecules with different mass.

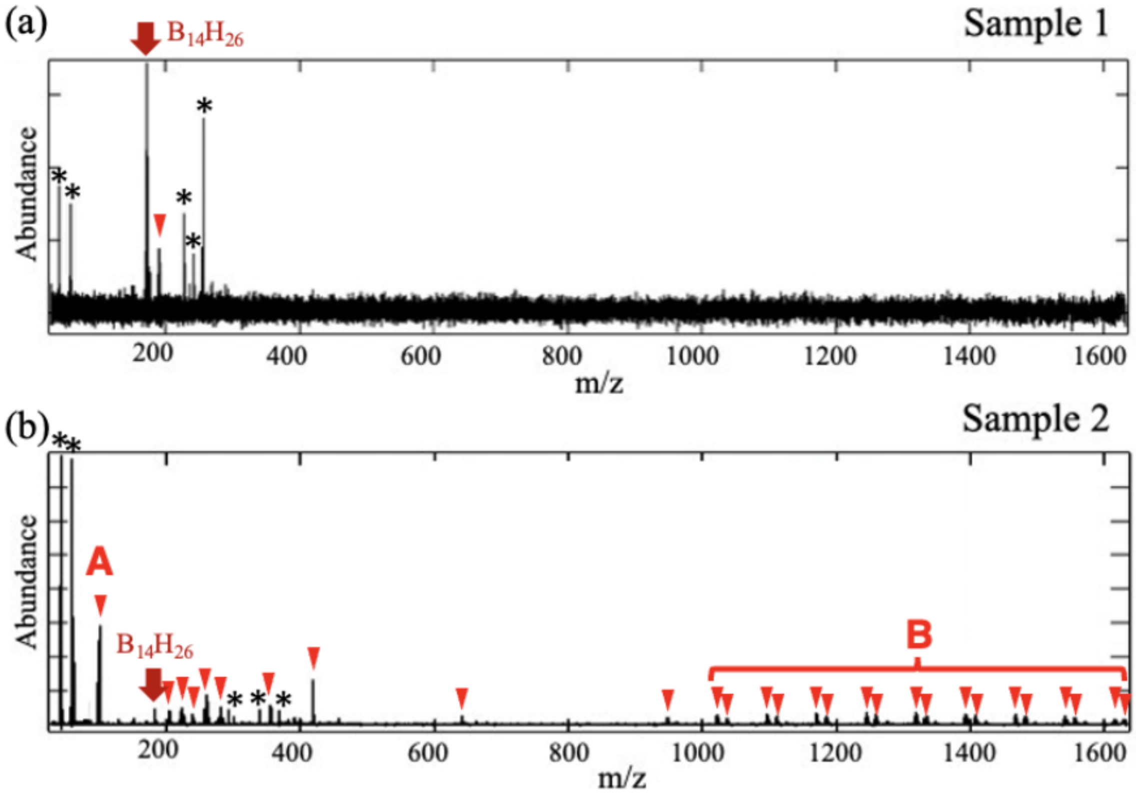

Figure. S 4: Two types of mass spectra of the solution samples that contained the vaporized species in the acetonitrile solvent: (a) sample 1-type and (b) sample 2-type. The borane peaks, as confirmed by the simulation, are indicated by red triangles (arrows for $B_{14}H_{26}$) and the others are labeled by asterisks. Unique mass patterns in (b) are noted by alphabets.

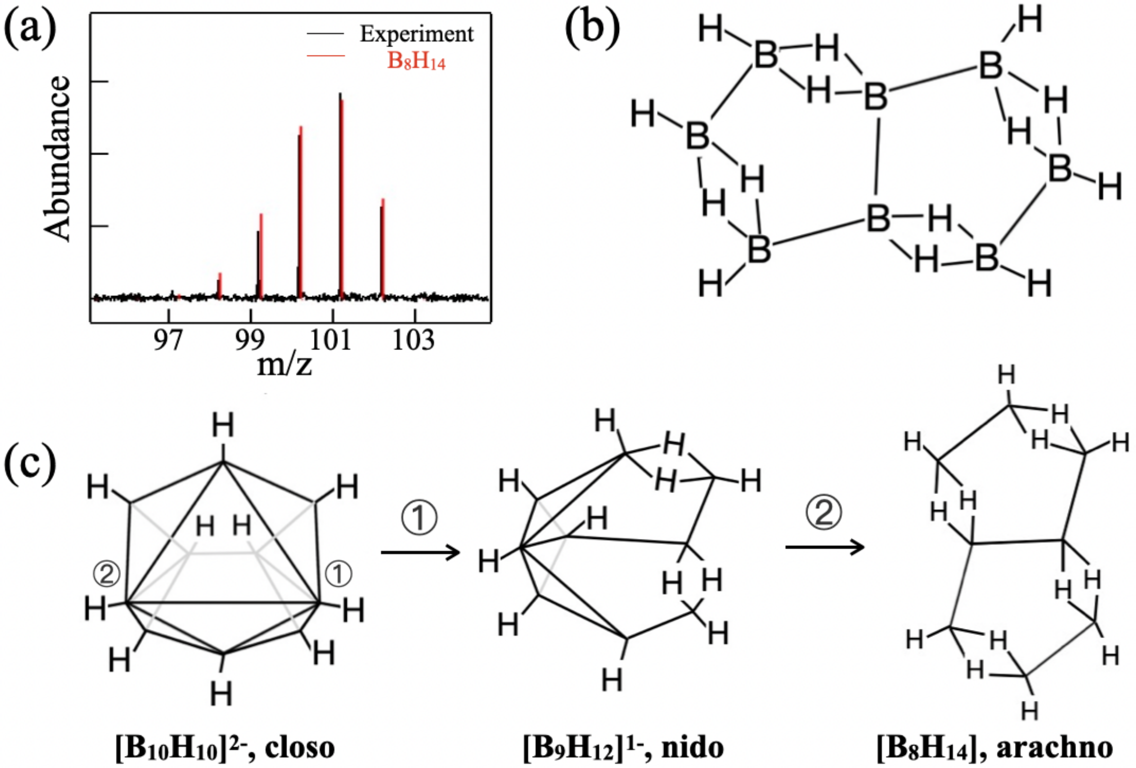

Figure. S 5: Detection of the B_8H_{14} cluster and the molecular structures. (a) Mass spectrum at m/z = 101, where m and z stand for mass and for charge number of ions, respectively. The simulated mass patterns of B_8H_{14} are indicated by red bars. (b) The atomic models of the B_8H_{14} isomers: a bicyclic structure of pentagons. (c) The derivation route in Wade's rule by removing the boron vertexes from the dodecahedron borane and adding an appropriate number of hydrogen atoms.

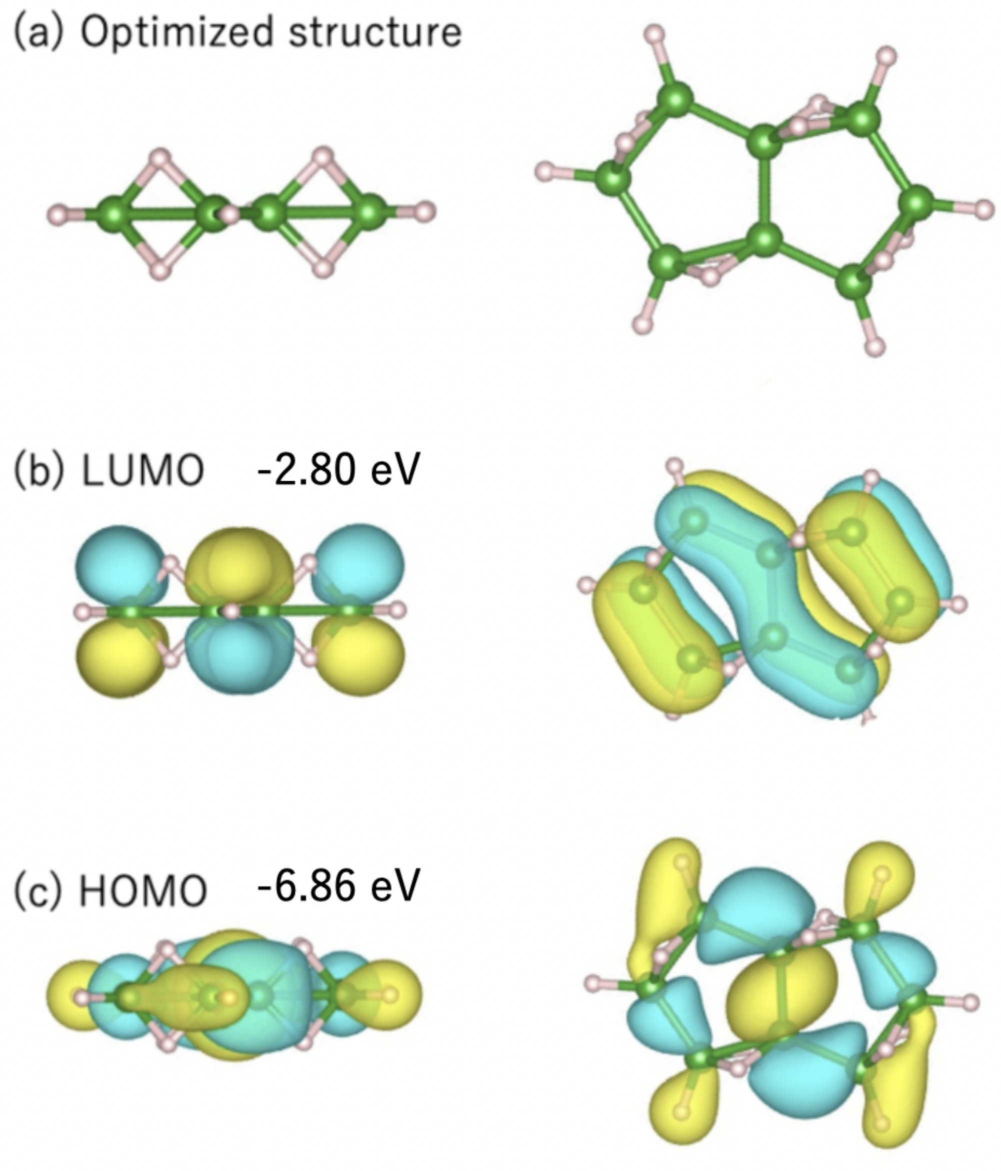

Figure. S 6: The calculated morphology of HOMO and LUMO for predicted borane molecule B_8H_{14}. Color corresponds to signs of the wave functions of orbitals.

3 Fourier transform infrared spectroscopy

To analyze the vaporized sample, collected from products of the ion-exchange reaction (Fig.S3), the solution (Fig.S3(c)) was dried by heating at 40°C. The resulting solid sample, Fig.S7(a), was then transferred into a gas cell (Fig.S7(b)) and subsequently pumped. FT-IR spectra of the vaporized samples were recorded after heating the sample at 45°C by electric (Joule) heaters.

FT-IR spectra of the HB sheet were obtained by the attenuated total reflectance (ATR) method. Powder samples of the HB sheets were dispersed on a prism holder and measured under the Ar gas environment at room temperature.

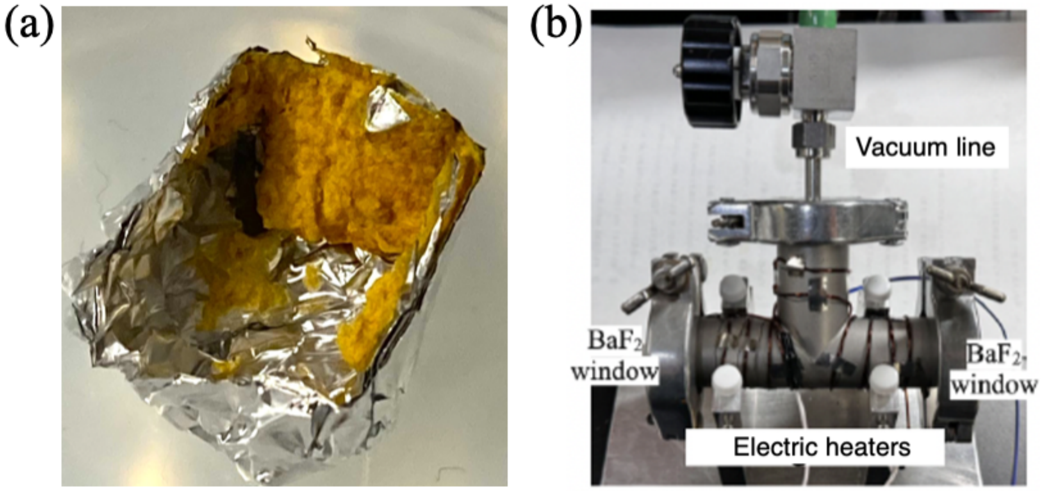

Figure. S 7: Photo of (a) the sample, dried after the vaporization, and (b) the gas cell in the FTIR system. The cell is equipped with BaF_2 windows and winded by electric heaters.

4 Stability of borane molecules in acetonitrile solution

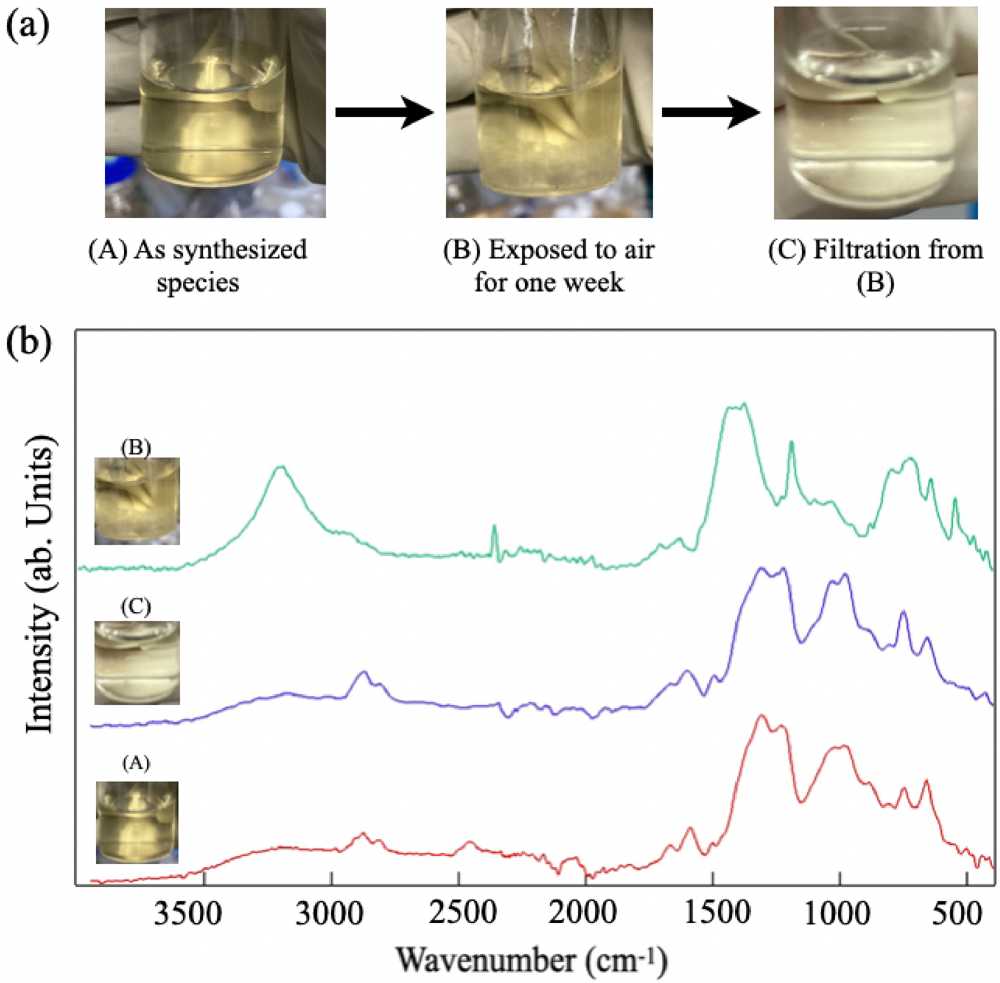

Figure. S 8: (a) Photo of boron species, synthesized and dissolved in acetonitrile solovent (A), exposed to air at room temperature for one week (B), and filtered to remove the percipipate (C). (b) The FT-IR spectra obtained from the 3 samples in (a).

The as-synthesized molecular species (A) (5 mg) was dissolved in 5 mL of acetonitrile, as shown in Fig. S8(a). The FT-IR spectrum was obtained using the ATR (attenuated total reflectance) method. The mixed solution was dropped onto the ATR crystal, dried under an argon atmosphere, and the FT-IR spectrum of the dried sample was recorded, as shown by the red curve in Fig. S8(b).

To evaluate the long-term stability of the sample, (A) was then placed in an ambient

environment (room temperature: 288–301 K) on a desk near a window for one week. After this exposure period, the sample showed visible turbidity at the bottom of the solution, as seen in Fig. S8(a) (labeled as B). The FT-IR spectrum for (B) was obtained in the same manner as (A) after a brief sonication treatment and is shown as the green curve in Fig. S8(b). The turbid solution (B) was subsequently filtered to separate the precipitate, yielding a clear filtrate, labeled as (C). The FT-IR spectrum of (C) is shown as the purple curve in Fig. S8(b).

A comparison of the spectra shows that (A) and (C) share similar spectral features, including characteristic peaks associated with B-H-B bonding (1350 cm^{-1}) and B-H stretching (1050 cm^{-1}, 700 cm^{-1}),[2,3,6,17] albeit with a slight reduction in intensity. In contrast, the spectrum of sample (B), which includes the white precipitate, shows prominent peaks characteristic of boric acid, such as B–O–H bending (1202 cm^{-1}), B–O stretching (1430 cm^{-1}), and O–H stretching (3250 cm^{-1}).[2,3,6,17] These changes likely result from the adsorption of oxygen (O_2) and water (H_2O) molecules onto the sample during air exposure, a phenomenon also reported for hydrogen boride sheets.[18–20]

5 Structure information from X-ray diffraction and NMR spectroscopy

The as-synthesized borane sample was dispersed on a silicon substrate for X-ray diffraction (XRD) analysis. The XRD patterns of the borane species, HB sheet, and boric acid provide insights into their structural characteristics, as illustrated in Fig. S9. The borane species (red curve) displays a broad, diffuse peak centered around $20 - 30^\circ$, indicative of an amorphous or poorly crystalline structure with limited long-range order. The absence of sharp peaks suggests that the borane exists predominantly in a non-crystalline state, likely as a molecular or cluster structure stabilized by hydrogen, rather than forming a regular crystal lattice. However, two small peaks at 27.2° and 27.2° hint at some degree of short-

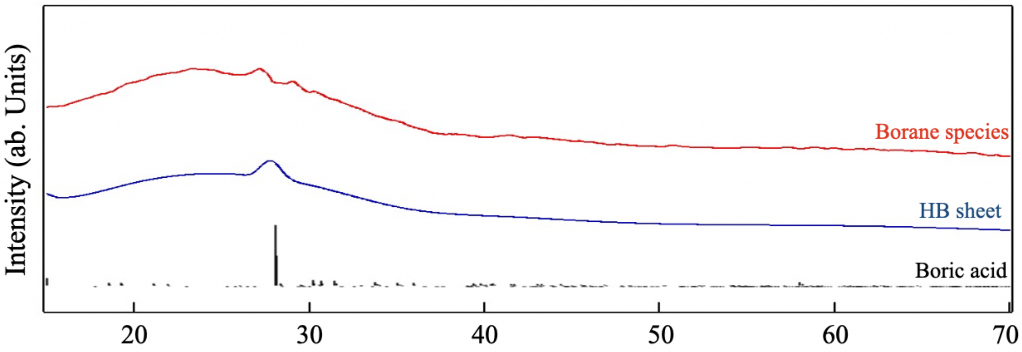

Figure. S 9: A collection of XRD patterns of the vaporized borane species (red curve), hydrogen boride/ HB sheet (blue curve) and the the calcuated pattern for the boric acid (black line).

range order within the otherwise amorphous structure. These minor peaks suggest that while the borane species $B_{14}H_{26}$ lacks extensive crystallinity, it may contain localized regions with distinct structural motifs that contribute to these specific reflections.

In comparison, the HB sheet (blue curve) also shows a broad peak in a similar range, implying partial structural similarity with the borane species but with subtle differences, potentially due to variations in layer stacking or degrees of partial crystallinity. The HB sheet additionally features a distinct peak at 28.1°, positioned between the two small peaks observed in the borane species, suggesting a more uniform or periodic arrangement. This peak at 28.1° may indicate layered stacking or a consistent planar ordering within the HB sheet structure. In contrast, the boric acid (black curve) exhibits sharp, well-defined peaks characteristic of a highly crystalline material, which are absent in both the borane species and HB sheet, highlighting their unique amorphous or semi-crystalline nature.

The ^{1}H NMR spectrum in Fig. S10 showed significant peaks at 1.06 (m), 1.98 (s), 2.60 (s), 3.30 (s), 3.46 (s), and 4.74 (s) ppm, which can be assigned to the terminal hydrogens bonded to the boron atoms (B–H) or the bridging hydrogens between the boron atoms (B–H–B).[21,22] Other signals around 2ppm can overlap with the strong signal originating from that of acetone in acetone-d_6. The spectrum also showed a broad peak at 3.75 ppm (br s $w_{1/2} \approx 640$ Hz), which likely correspond to B–H–B bonds,[21,22] where the broadness implies

the unique molecular structure of the borane compounds, possibly originating from the B_2H_2 clusters via B–B bonds. The sample may be contaminated with decomposed products from the ion-exchange resin, such as 4-vinylbenzenesulfonate salts, used in the fabrication procedure. The decoupled ^{11}B NMR spectrum in Fig. S11 showed a signal at 21.1 ppm, like that of the diborane B_2H_6 molecule,[23] implying the sample may consist of diborane-like components. Its slight broadening can be due to the rapid exchange between terminal H and bridging H between B and B atoms, or it could indicate the possible weak B–B coupling in an intramolecular manner. There appears to be another broad signal around –5 ppm, although overlapping with the reference $^{11}BF_3OEt_2$. The possible signal might reflect the B atoms with a different electronic environment, possibly based on B-B bond formation.

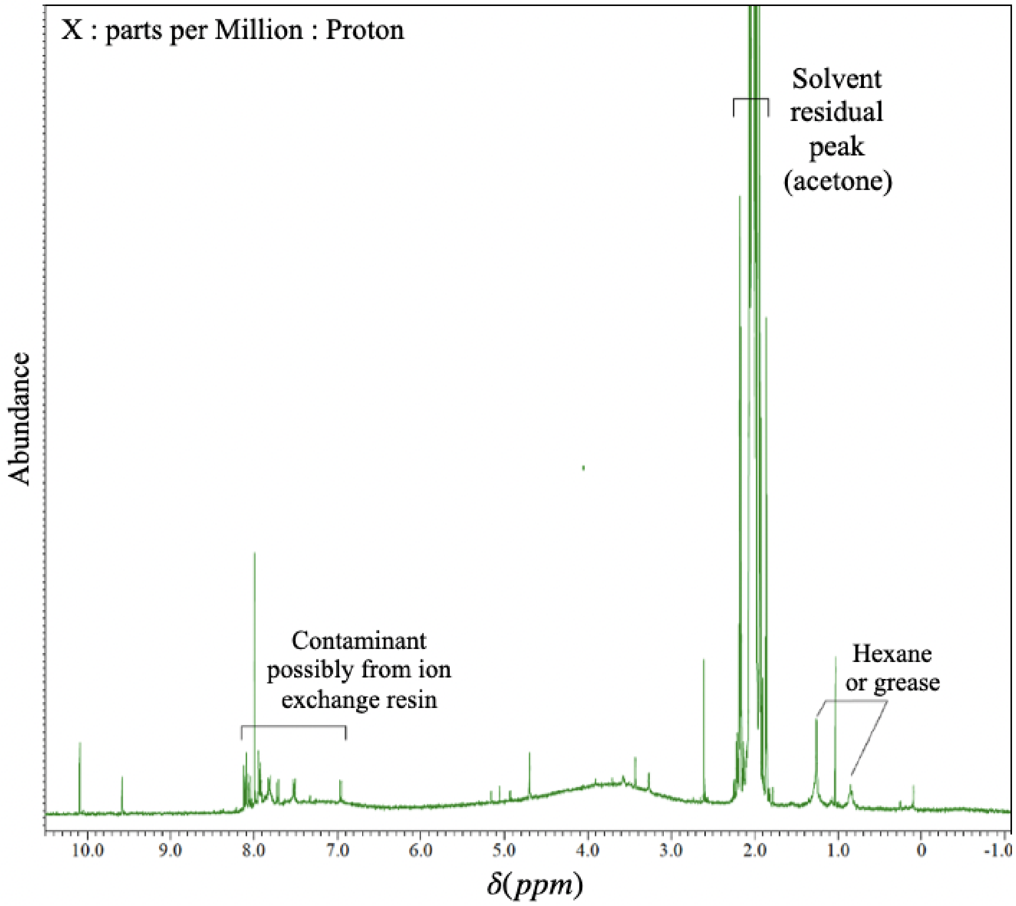

Figure. S 10: ^{1}H NMR of the borane sample in acetone-d_6 acquired on a JEOL ECS-400 spectrometer (400 MHz).

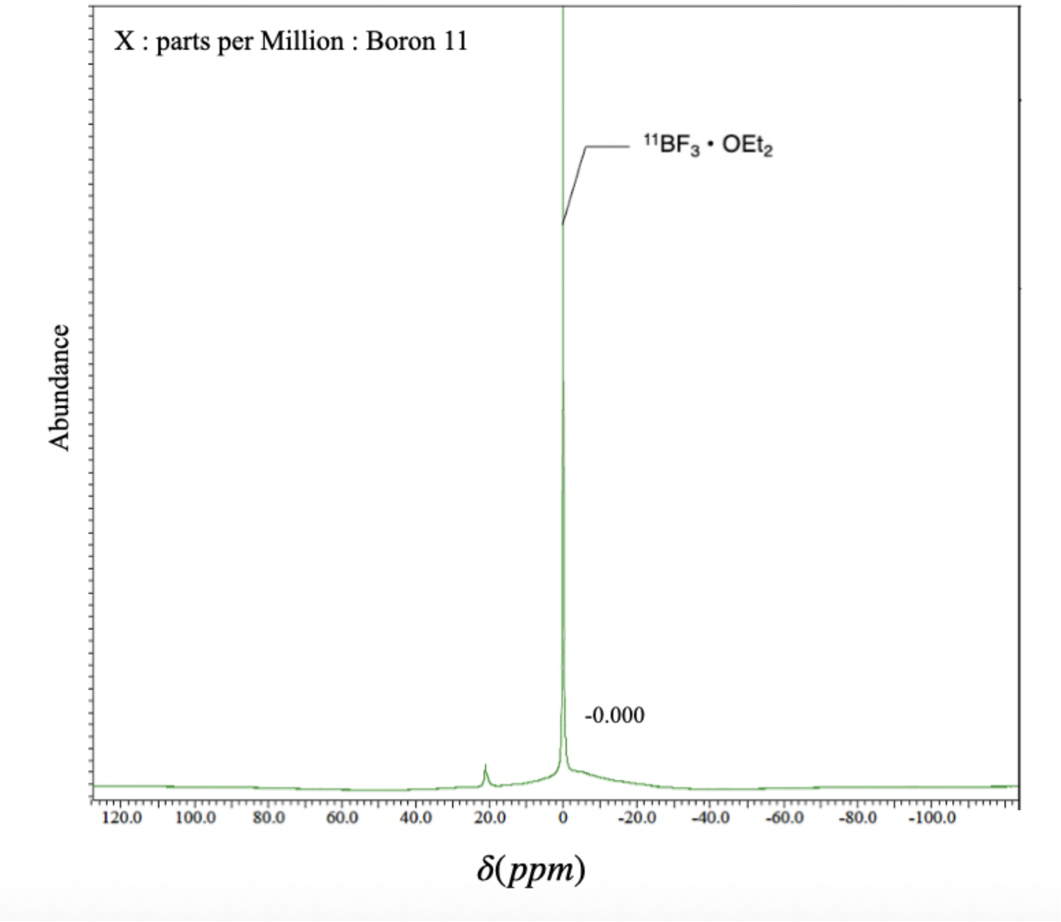

Figure. S 11: ^{11}B NMR spectrum of the borane sample in acetone-d_6 acquired on a JEOL ECS-400 spectrometer (128 MHz) with internal reference to $^{11}BF_3OEt_2$ in a sealed capillary tube.

6 Bicyclic borane molecule with a octagon and a fulvene-like heptagon

The $B_{14}H_{26}$ molecule, discovered in the present research, is classified in the Q-type in a series of borane, based on Wade's rule. Starting from the dodecahedron borane, one can derive the $B_{14}H_{26}$ structure as a bicyclic molecule of fulvene-like heptagons or of octagons (Fig. S5). The molecular structure is consistent to the two-dimensional boron structure in the HB sheet and the $YCrB_4$ crystal that contains the heptagon boron (Fig.S1). As an isomer of the $B_{14}H_{26}$ molecule, one can also consider the intermediate structure that corresponds to a bicyclic molecule with a composition of a fulvene-like heptagon and an octagon, as illustrated in Fig. S12(a). Similar to Fig. S5, a similar derivation can be made from the dodecahedron borane, as given in Fig. S12(b). The molecular orbital of the intermediate $B_{14}H_{26}$ is depicted in the Fig. S13. Energy level of HOMO (LUMO) for the intermediate $B_{14}H_{26}$ is located at -6.94 (-1.96) eV referred to the vacuum level. It is of note that the theoretically optimized molecule with a composition of two fulvene-like heptagons or two octagons (Fig.1) belong to the C_i symmetry, while that of one fulvene-like heptagon and one octagons (Fig.S13(a)), belongs to the C_1 symmetry. S

7 The Hückeloid model for cyclic borane molecules

Properties of borane have been historically investigated through a comparison with hydrocarbon. A carbon (C) atom has four valence orbitals (2s, $2p_x$, $2p_y$, $2p_z$) and four valence electrons, making the well-defined bonding scheme that have been described in terms of the octet rule. An atom of boron (B), located next to C in the periodic table, has also four valence orbitals (2s, $2p_x$, $2p_y$, $2p_z$) but three valence electrons. The situation provides a degree of freedom in making varieties of bonding scheme for boron. An example is the three-center two-electron (3c-2e) bond in borane, where the boron atoms have been considered to mimic

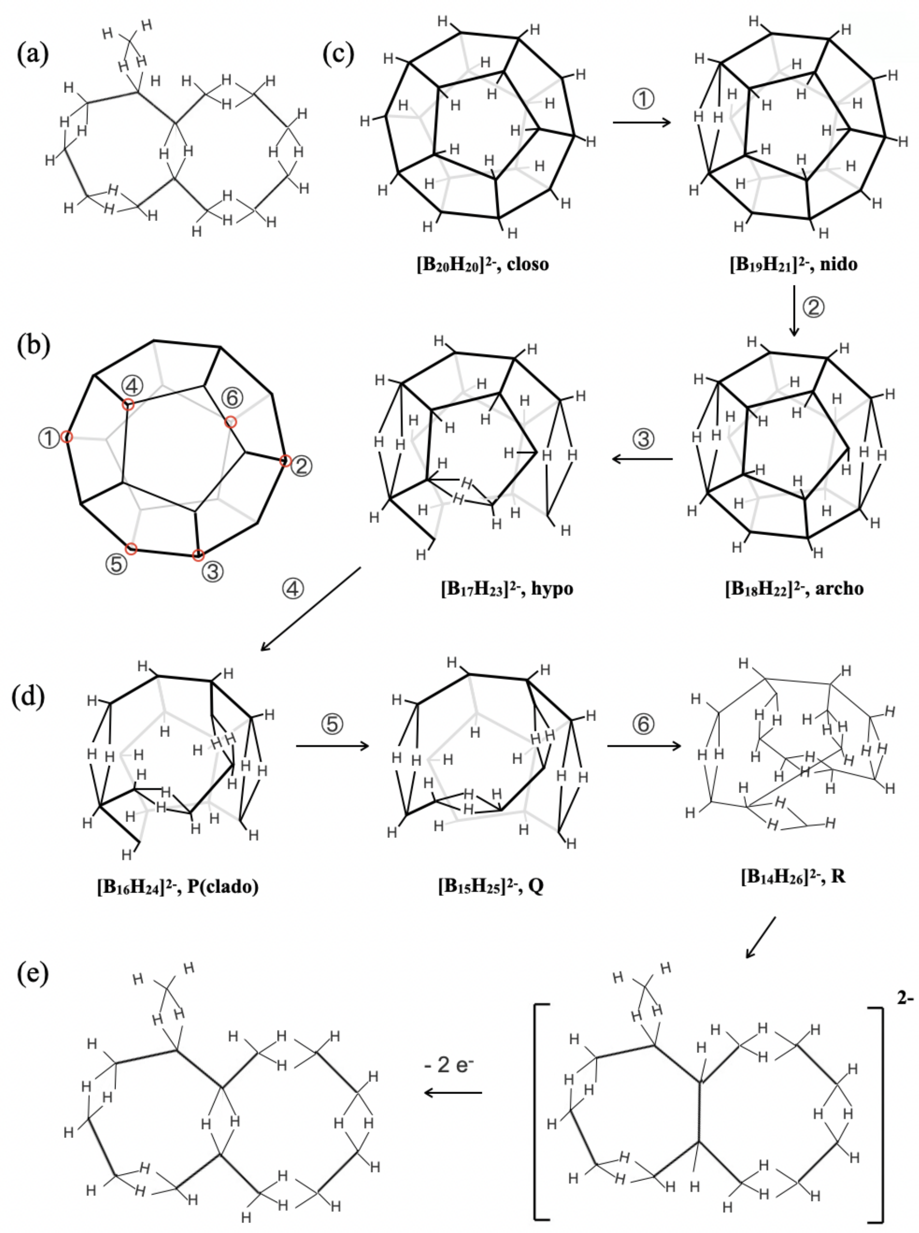

Figure. S 12: Bicyclic borane molecule, $B_{14}H_{26}$, with an octagon and a fulvene-like heptagon. (a) Molecular structure. (b) Six sites of the boron vertex in the dodecahedron borane, labeled by numbers. (c) The derivation route from the dodecahedron borane, based on Wade's rule.

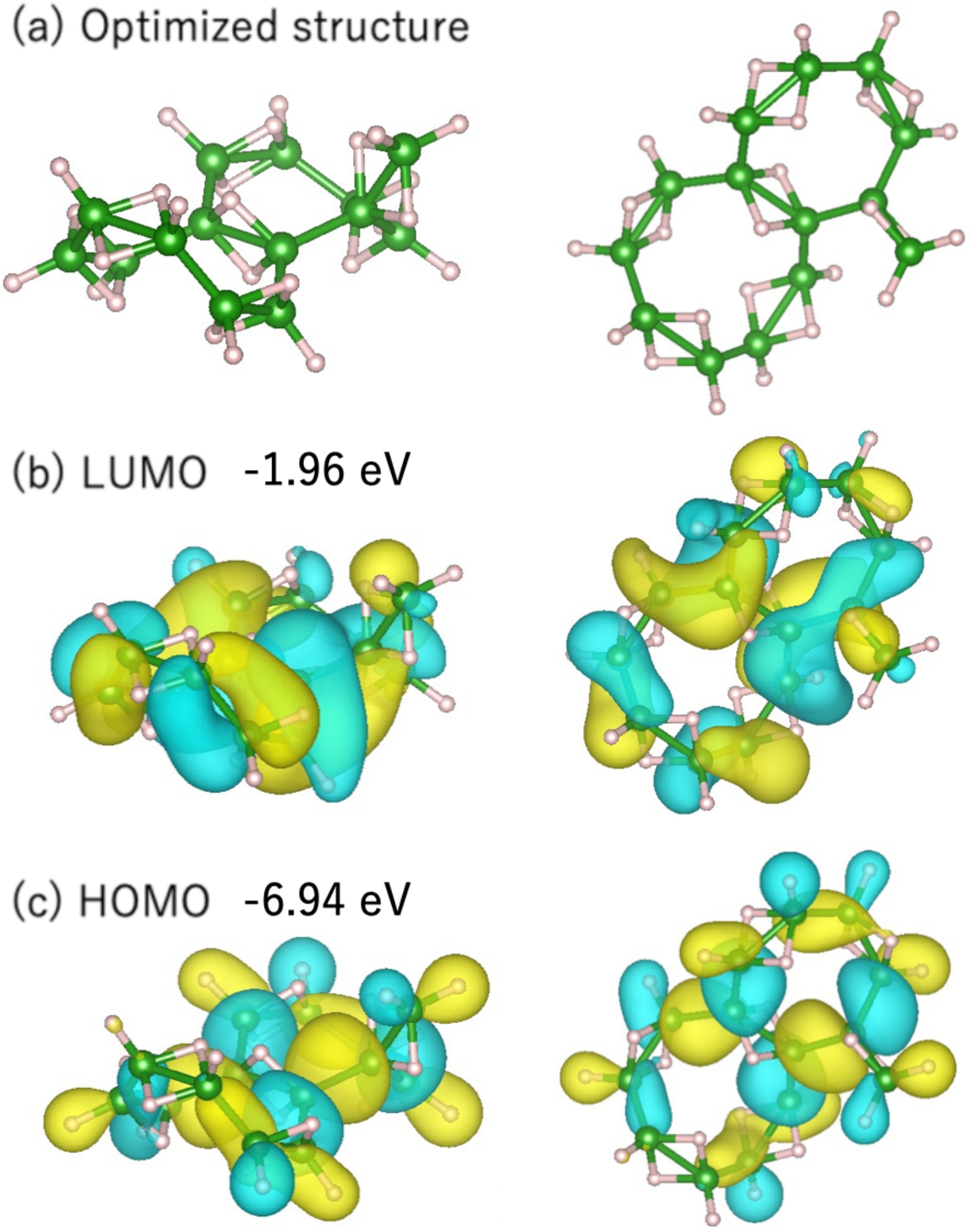

Figure. S 13: The calculated morphology of HOMO and LUMO for predicted borane molecule $B_{14}H_{26}$ composing of a fulvene-like heptagon and an octagon. Color corresponds to signs of the wave functions of orbitals.

the carbon atom in an attempt to obey the octet rule. One may consider, for instance, that diborane as ethylene since the BH fragment is electronically equivalent to a C atom. The argument has extended to the larger molecules, including the cyclic ones, and, recently, the Hückeloid model was developed in analogy to the Hücklel model.[24,25]

A molecular orbital theory of the Hückel model is based on a simple molecular orbit Hückel's rule that explains the aromaticity in terms of the 4n+2 π-electrons. In the Hückeloid model, a borane molecule is regarded as an unsaturated hydrocarbon by replacing the double bonds (-C=C-) with 3c-2e (-BH_2B-) bonds. The tight-binding Hamiltonian for the Kekule structure $\kappa(S)$ of a planar molecule is given for the π-electrons as follows:[24,25]

$$H_{B(\kappa(S))} = t \sum_{a \sim b}^{S} T_{a,b} + t_b \sum_{\{a,b\}}^{\pi-\kappa(S)} (T_{ab,a} + T_{ab,b}) + \epsilon_b \sum_{\{a,b\}}^{\pi-\kappa(S)} T_{ab,ab} \tag{1}$$

where a and b indicates boron (B) atoms. The label, ab, means an H_2 orbitals midways between boron atoms a and b. The operators, $T_{a,b}$ and $T_{ab,x}$, are defined as follows:

$$T_{a,b} \equiv \sum_{s}^{\in\{\alpha,\beta\}} (c^{+}_{a,s} c_{b,s} + c^{+}_{b,s} c_{a,s}) \tag{2}$$

$$T_{ab,x} \equiv \sum_{s}^{\in\{\alpha,\beta\}} (c^{+}_{ab,s} c_{x,s} + c^{+}_{x,s} c_{ab,s}) \tag{3}$$

where $c^{+}_{x,s}$ ($c^{+}_{ab,s}$) and $c_{x,s}$ ($c_{ab,s}$) are creation and annihilation operators, respectively, for B orbitals (H_2 orbitals) at a B site x with a spin $s \in \{\alpha, \beta\}$.

The first term in Eq.(1) corresponds to the Hückel model for S with the electron-transfer parameter t between any adjacent $2p_z$ orbitals of boron. The second term in Eq. (1) is responsible for bridging $-BH_2B-$ bonds with the electron-transfer parameter t_b between a $2p_z$ orbital of boron and an adjacent H_2 orbital. The third term in Eq. (1) has an on-site energy ϵ_b for the H_2 component with respect to the energy of boron orbital. The previous research has obtained good matching in the energy structures with the Hückel model and the Hückeloid model on a simple borane molecules, such as benzene-type and pentaene-type.[24,25]

Adopting the parameters from the reference,[24,25] we set $2t{=}t_b$ and ϵ_b = - 0.2 $|t|$. Then, we made calculations of the Hückel model and the Hückeloid model for isomers of the bicyclic $B_{14}H_{26}$ molecule and the related molecule of B_8H_{14}(Fig.S5,S6). The results are shown in Fig. S14. One finds the good matching between the Hückel model and the Hückeloid model, as in the case of the reference.[24,25] The consistency supports existence of the bicyclic borane molecules, based on a fullvene-like heptagon, an octagon, and/or a pentagon, proposed in this research.

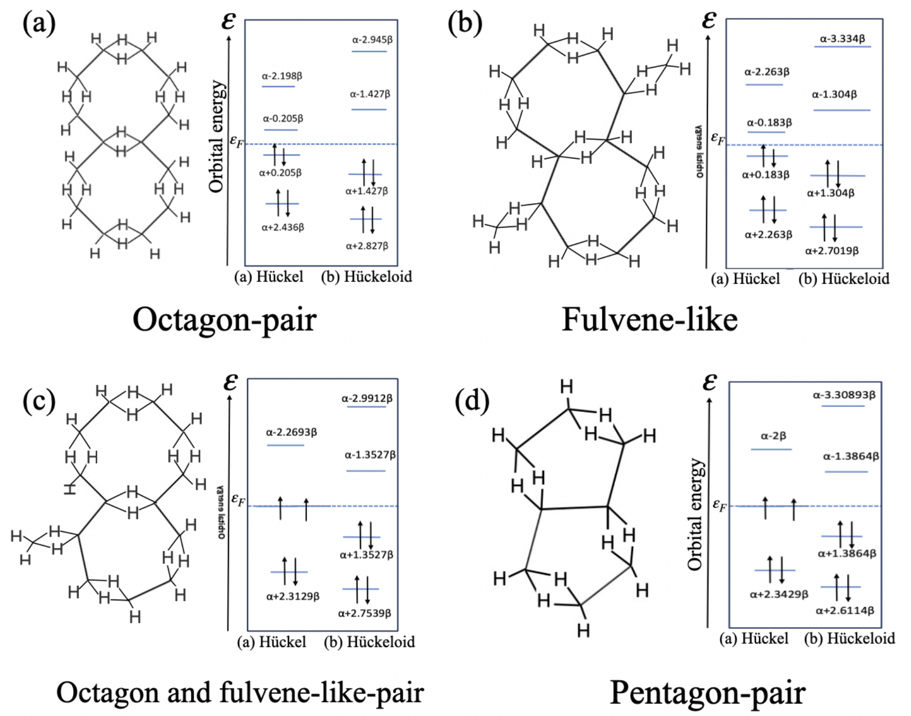

Figure. S 14: Application of the Hückel model and the Hückeloid model for isomers of (a-c) the bicyclic $B_{14}H_{26}$ molecule, and (d) pentagon B_8H_{14} molecule. The orbital energies of molecular orbitals, including HOMO and LUMO, are given with occupations of spin-dependent electrons.

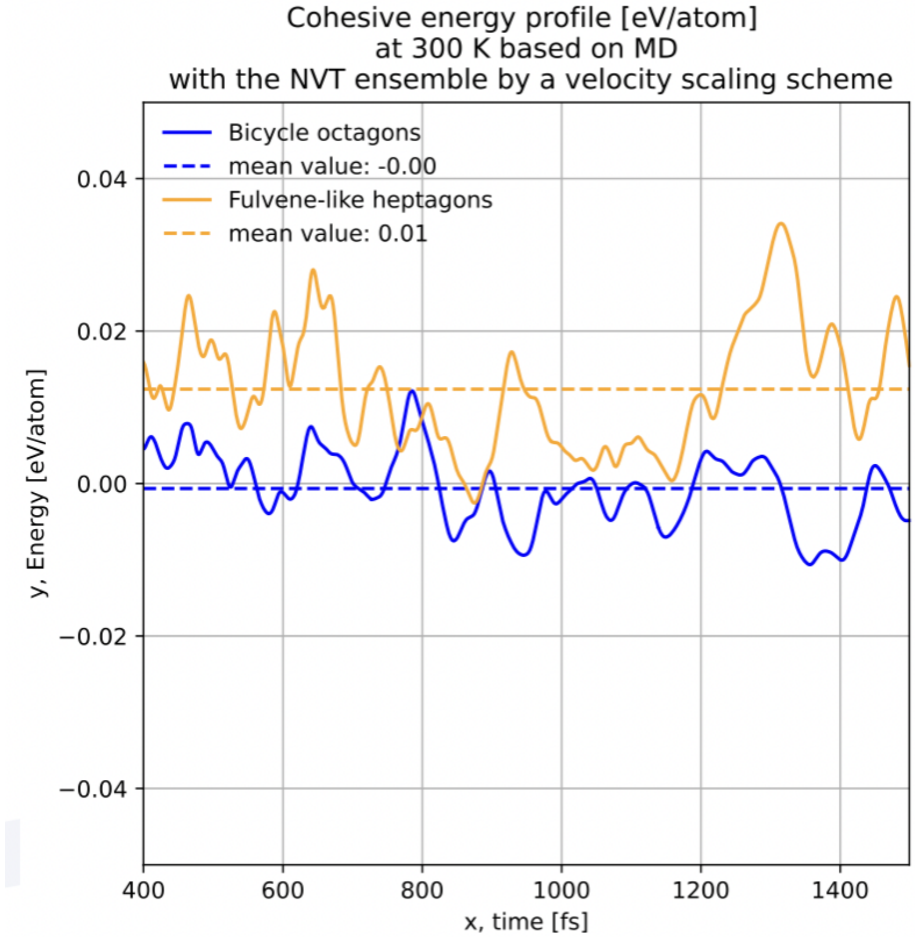

Figure. S 15: Cohesive energy profile $E_{cohesive}$ (in eV/atom) of two $B_{14}H_{26}$ molecular structures, bicycle octagons (blue) and fulvene-like heptagons (orange), calculated using molecular dynamics (MD) at 300 K in the NVT ensemble with velocity scaling. The cohesive energy oscillates around the mean values of -0.00 eV/atom and 0.01 eV/atom for the octagonal and heptagonal structures, respectively.

8 Cohesive energies analysis on the bicyclic $B_{14}H_{26}$ molecule

We calculated the cohesive energy, $E_{cohesive}$ [eV/atom], to evaluate the stability of each $B_{14}H_{26}$ molecule. Fig. S15 demonstrates the calculated results, showing that the cohesive energies of both molecular structures oscillate around their respective mean values. However, these values are relatively small compared to the thermal vibrational energy at 300 K (approximately 25.8 meV), making it challenging to observe a substantial difference in cohesive energy per atom between the two configurations.

9 Methods

Powder X-Ray diffraction Powder X-Ray diffraction patterns were collected using a Rigaku SmartLab X-ray diffractometer equipped with a CuKα radiation source ($\lambda = 1.5418$ Å) operated at 40 kV and 30 mA. Samples were scanned over a 2θ range of 10˘80°.

Nuclear Magnetic Resonance (NMR) Spectroscopy The proton (^{1}H) and boron (^{11}B) nuclear magnetic resonance (NMR) spectrum were recorded on a JEOL ECS-400 (^{1}H: 400 MHz, ^{11}B: 128 MHz) spectrometer. ^{1}H NMR spectra in acetone-d_6 (Wako Pure Chemical Industries) were referenced to the solvent resonance. The decoupled ^{11}B NMR spectrum in acetone-d_6 was referenced internally to $BF_3 \cdot OEt_2$ (Wako Pure Chemical Industries), used in a sealed glass capillary.

Details of the first-principles calculation The cohesive energy was calculated by using OpenMX v.3.9. as following equation:

$$E_{cohesive}[eV/atom] = \frac{E_{total} - 2 \times E_{isolated}^{average}}{N}[eV/atom] \quad (4)$$

where E_{total} represents the total energy of two $B_{14}H_{26}$ molecules, $E_{isolated}^{average}$ is the energy of a single $B_{14}H_{26}$ molecule, and N is the total number of atoms, calculated as 2×(14+26) = 80. The cohesive energy calculation was performed in the canonical ensemble (NVT) at

300 K with a velocity scaling scheme,[26] including van der Waals interactions through the DFT-D3 correction.[27]

Supplementary References

(1) Matsuda, I.; Wu, K. *2D boron: boraphene, borophene, boronene*; Springer, 2021.

(2) Zhang, X.; Tsujikawa, Y.; Tateishi, I.; Niibe, M.; Wada, T.; Horio, M.; Hikichi, M.; Ando, Y.; Yubuta, K.; Kondo, T.; Matsuda, I. Electronic Topological Transition of 2D Boron by the Ion Exchange Reaction. *J. Phys. Chem. C* **2022**, *126*, 12802–12808.

(3) Nishino, H.; Fujita, T.; Cuong, N. T.; Tominaka, S.; Miyauchi, M.; Iimura, S.; Hirata, A.; Umezawa, N.; Okada, S.; Nishibori, E.; others Formation and characterization of hydrogen boride sheets derived from MgB_2 by cation exchange. *J. Am. Chem. Soc.* **2017**, *139*, 13761–13769.

(4) Cuong, N. T.; Tateishi, I.; Cameau, M.; Niibe, M.; Umezawa, N.; Slater, B.; Yubuta, K.; Kondo, T.; Ogata, M.; Okada, S.; Matsuda, I. Topological Dirac nodal loops in nonsymmorphic hydrogenated monolayer boron. *Phys. Rev. B* **2020**, *101*, 195412.

(5) Tateishi, I.; Zhang, X.; Matsuda, I. Electronic Structures of Polymorphic Layers of Borophane. *Molecules* **2022**, *27*, 1808.

(6) Zhang, X.; Hikichi, M.; Iimori, T.; Tsujikawa, Y.; Yuan, M.; Horio, M.; Yubuta, K.; Komori, F.; Miyauchi, M.; Kondo, T.; Matsuda, I. Accelerated Synthesis of Borophane (HB) Sheets through HCl-Assisted Ion-Exchange Reaction with $YCrB_4$. *Molecules* **2023**, *28*, 2985.

(7) Zhang, X.; Tsujikawa, Y.; Yamaguchi, K.; Miyamoto, M.; Horio, M.; Yubuta, K.; Ando, H.; Yuan, M.; Ozawa, K.; Sugiyama, K.; Kondo, T.; Matsuda, I. Millimeter-

scale growth of $YCrB_4$ single crystals and observation of the metallic surface state. *Phys. Rev. Mater.* **2024**, *8*, 054001.

(8) Momma, K.; Izumi, F. *VESTA3* for three-dimensional visualization of crystal, volumetric and morphology data. *J. Appl. Crystallogr.* **2011**, *44*, 1272–1276.

(9) Josuran, R. ProtPI, Mass Spectrum Simulator. `https://www.protpi.ch/calculator/MassSpecSimulator`.

(10) Hagemann, H. Boron Hydrogen Compounds: Hydrogen Storage and Battery Applications. *Molecules* **2021**, *26*.

(11) Ghosh, S.; Noll, B. C.; Fehlner, T. P. Borane Mimics of Classic Organometallic Compounds: [(Cp*Ru)B8H14(RuCp*)]0,+, Isoelectronic Analogues of Dinuclear Pentalene Complexes. *Angew. Chem., Int. Ed.* **2005**, *44*, 6568–6571.

(12) Boucher, B.; Ghosh, S.; Halet, J.-F.; Kahlal, S.; Saillard, J.-Y. Bonding and electronic structure of $Cp*_2 Ru_2(B_8H_{14})$, a metallaborane analogue of dinuclear pentalene complexes. *J. Organomet. Chem.* **2012**, *721-722*, 167–172, Special issue on Boron: Dedicated to Professor Thomas P. Fehlner on the occasion of his 75th Birthday.

(13) Ghosh, S.; Noll, B. C.; Fehlner, T. P. Borane Mimics of Classic Organometallic Compounds: $[(Cp*Ru)B_8H_{14}(RuCp*)]^{0,+}$, Isoelectronic Analogues of Dinuclear Pentalene Complexes. *Angew. Chem., Int. Ed. Engl.* **2005**, *44*, 6568–6571.

(14) King, R. B. Defective Vertices in arachno Borane Networks. *Inorg. Chem.* **2003**, *42*, 3412–3415, PMID: 12767175.

(15) Kiremire, E. M. R.; Lule, I. Categorization of Boranes Into Clan Series. *Int. J. Chem.* **2020**, *12*, 107–151.

(16) Ando, Y.; Nakashima, T.; Yin, H.; Tateishi, I.; Zhang, X.; Tsujikawa, Y.; Horio, M.;

Cuong, N. T.; Okada, S.; Kondo, T.; others Prediction of a Cyclic Hydrogenated Boron Molecule as a Promising Building Block for Borophane. *Molecules* **2023**, *28*, 1225.

(17) Hikichi, M.; Takeshita, J.; Noguchi, N.; Ito, S.-i.; Yasuda, Y.; Ta, L. T.; Rojas, K. I. M.; Matsuda, I.; Tominaka, S.; Morikawa, Y.; others Controlling Photoinduced H2 Release from Freestanding Borophane Sheets Under UV Irradiation by Tuning B–H Bonds. *Adv. Mater. Interfaces* **2023**, *10*, 2300414.

(18) Rojas, K. I. M.; Cuong, N. T.; Nishino, H.; Ishibiki, R.; Ito, S.-i.; Miyauchi, M.; Fujimoto, Y.; Tominaka, S.; Okada, S.; Hosono, H.; Arboleda, N. B.; Kondo, T.; Morikawa, Y.; Hamada, I. Chemical stability of hydrogen boride nanosheets in water. *Commun. Mater.* **2021**, *2*, 81.

(19) Ito, S.-i.; Hikichi, M.; Noguchi, N.; Yuan, M.; Kang, Z.; Fukuda, K.; Miyauchi, M.; Matsuda, I.; Kondo, T. Effective treatment of hydrogen boride sheets for long-term stabilization. *Phys. Chem. Chem. Phys.* **2023**, *25*, 15531–15538.

(20) Zhang, X.; Miyamoto, M.; Yuan, M.; Tsujikawa, Y.; Yamaguchi, K.; Horio, M.; Ozawa, K.; Yubuta, K.; Kondo, T.; Matsuda, I. Fermi Edge of Semimetallic Borophane Sheets and its Reduction by a Porous Structure. *The Journal of Physical Chemistry Letters* **2024**, *15*, 9349–9355.

(21) Wehmschulte, R. J.; Diaz, A. A.; Khan, M. A. Unsymmetrical 9-Borafluorenes via Low-Temperature C- H Activation of m-Terphenylboranes. *Organometallics* **2003**, *22*, 83–92.

(22) Hübner, A.; Diefenbach, M.; Bolte, M.; Lerner, H.-W.; Holthausen, M. C.; Wagner, M. Confirmation of an early postulate: BCB two-electron-three-center bonding in organo (hydro) boranes. *Angewandte Chemie (International ed. in English)* **2012**, *51*, 12514–12518.

(23) Hermanek, S. Boron-11 NMR spectra of boranes, main-group heteroboranes, and substituted derivatives. Factors influencing chemical shifts of skeletal atoms. *Chemical reviews* **1992**, *92*, 325–362.

(24) Yates, K. *Hückel molecular orbital theory*; Elsevier, 2012.

(25) Klein, D. J.; Ferrer, M.; Elguero, J.; Bytautas, L.; Oliva-Enrich, J. M. Hückeloid model for planar boranes. *Theor. Chem. Acc.* **2021**, *140*, 55.

(26) Woodcock, L.-V. Isothermal molecular dynamics calculations for liquid salts. *Chemical Physics Letters* **1971**, *10*, 257–261.

(27) Grimme, S.; Antony, J.; Ehrlich, S.; Krieg, H. A consistent and accurate ab initio parametrization of density functional dispersion correction (DFT-D) for the 94 elements H-Pu. *The Journal of chemical physics* **2010**, *132*.
