# Supplementary material for: Discovery of bicyclic borane molecule B14H26
Source: Commun Chem. 2025 Jan 16;8:14. doi: 10.1038/s42004-025-01409-1 (PMC11739403; doi:10.1038/s42004-025-01409-1)
Supplement: Supplementary file 2 — Description of Additional Supplementary Data Files [file 42004_2025_1409_MOESM2_ESM.pdf]

Description of Additional Supplementary Files

File name: Supplementary Data 1
Description: The optimized structure of Octagon $B_{14}H_{26}$ (in XYZ format) , calculated by the first principles calculation in the present study.
Researchers can view or analyze this file using any standard molecular visualization or computational chemistry software to examine the detailed bonding and spatial arrangement of the $B_{14}H_{26}$ octagonal cluster.

File name: Supplementary Data 2
Description: The optimized structure of Fulvene-like $B_{14}H_{26}$ (in XYZ format) , calculated by the first principles calculation in the present study.
Researchers can view or analyze this file using any standard molecular visualization or computational chemistry software to examine the detailed bonding and spatial arrangement of the Fulvene-like $B_{14}H_{26}$ cluster.

File name: Supplementary Data 3
Description: The optimized structure of Pentagon-pair B_8H_{14} (in XYZ format) , calculated by the first principles calculation in the present study.
Researchers can view or analyze this file using any standard molecular visualization or computational chemistry software to examine the detailed bonding and spatial arrangement of the Pentagon-pair B_8H_{14} cluster.

The optimized structure (xyz file) of Pentagon-pair B_8H_{14}

File name: Supplementary Data 4
Description: The optimized structure of Octagon and fulvene-like-pair $B_{14}H_{26}$ (in XYZ format) , calculated by the first principles calculation in the present study.
Researchers can view or analyze this file using any standard molecular visualization

or computational chemistry software to examine the detailed bonding and spatial arrangement of the Octagon and fulvene-like-pair $B_{14}H_{26}$ cluster.
